# Nanocarrier-Based Drug Delivery Systems for Lung Cancer: A Systematic Review and Meta-Analysis of Preclinical Studies

**DOI:** 10.3390/arm94040050

**Published:** 2026-07-24

**Authors:** Pranvera Breznica Selmani, Arlinda Daka Grapci, Blerina Koshi, Zana Sllamniku Dalipi, Rozafa Koliqi

**Affiliations:** 1 Department of Pharmaceutical Chemistry, Pharmacy Division, Faculty of Medicine, University of Prishtina, 10000 Prishtina, Kosovo; pranvera.breznica@uni-pr.edu; 2 Department of Clinical Pharmacy and Biopharmacy, Pharmacy Division, Faculty of Medicine, University of Prishtina, 10000 Prishtina, Kosovo; arlinda.daka@uni-pr.edu; 3 Department of Pharmaceutical Technology, Pharmacy Division, Faculty of Medicine, University of Prishtina, 10000 Prishtina, Kosovo; blerina.koshi@uni-pr.edu; 4 Department of Dentistry, Faculty of Medicine, University of Prishtina, 10000 Prishtina, Kosovo; zana.sllamniku@uni-pr.edu

**Keywords:** drug delivery systems, lung cancer, nanoparticles, targeted therapy, chemotherapy, combination therapy, tumor inhibition, meta-analysis, preclinical models

## Abstract

**Highlights:**

**What are the main findings?**

- DDS-based chemotherapy significantly reduced tumor volume compared with corresponding free-drug treatments in preclinical lung cancer models.
- Targeted delivery systems, nanoparticle-based platforms, and combination therapies showed larger average effect in subgroup analyses, although these findings should be interpreted cautiously because of heterogeneity and potential confounding by formulation and study-design characteristics.

**What are the implications of the main findings?**

- The design and targeting strategy of drug delivery systems are important determinants of therapeutic success in lung cancer.
- These findings support the further standardized and translationally oriented preclinical development of nanocarrier-based therapies, but they do not establish clinical efficacy.

**Abstract:**

Drug delivery systems (DDS) may improve the therapeutic performance of chemotherapy in lung cancer, but their preclinical efficacy has not been quantitatively synthesized. We conducted a systematic review and meta-analysis of controlled in vivo mouse studies evaluating DDS-based chemotherapeutic formulations for lung cancer. Databases were searched from inception to 15 February 2025, and methodological quality was assessed using the SYRCLE risk-of-bias tool. Thirty studies comprising 47 experiments were included. Compared with corresponding free-drug treatments, DDS-based chemotherapy significantly reduced tumor volume (WMD −310.67 mm^3^; 95% CI: −375.51 to −245.83; *p* < 0.001), although substantial heterogeneity was observed. Both targeted and non-targeted DDS were associated with tumor growth inhibition, and targeted formulations showed a larger average reduction; however, this finding should be interpreted in light of differences in formulation properties, tumor models, and treatment protocols. Nanoparticle, liposomal, and micellar platforms all demonstrated significant antitumor effects, while combination DDS and docetaxel- or cisplatin-based systems showed large effects in subgroup analyses with variable sample sizes. These findings support the continued development of DDS-based chemotherapy for lung cancer, but standardized reporting of nanocarrier characterization, pharmacokinetics, biodistribution, toxicity, and rigorous animal-study design is required to improve reproducibility and translational relevance.

## 1. Introduction

Lung cancer remains one of the leading causes of cancer-related mortality worldwide and continues to represent a major public health challenge [1]. The disease is characterized by uncontrolled proliferation of malignant cells within the lung and a high propensity for local invasion and distant metastasis, which accounts for most lung cancer-related deaths [1]. Although the incidence is higher among older adults, lung cancer can also affect younger individuals due to genetic susceptibility, tobacco exposure, environmental pollutants, and occupational risk factors [2,3,4]. Current treatment strategies for lung cancer include surgery, radiotherapy, chemotherapy, targeted therapies, and immunotherapy, with treatment selection depending on tumor stage, histological subtype, and molecular characteristics [5]. Despite significant therapeutic advances, chemotherapy remains an important component of treatment for many patients. However, the clinical efficacy of conventional chemotherapeutic agents is often limited by poor tumor selectivity, rapid systemic distribution, short circulation time, dose-limiting toxicity, and the development of drug resistance [6]. In addition, the complex tumor microenvironment, heterogeneous vascularization, impaired drug penetration, and metastatic spread further reduce treatment effectiveness and contribute to disease progression. For patients with pleural or peritoneal involvement, locoregional administration approaches such as intrapleural or intraperitoneal chemotherapy have been used alongside systemic treatment to improve local disease control and survival outcomes [7]. Nevertheless, achieving adequate drug concentrations at the tumor site while minimizing systemic toxicity remains a major challenge in lung cancer therapy. Drug delivery systems (DDS) have emerged as promising tools to address these limitations by improving the pharmacokinetic and pharmacodynamic properties of anticancer agents [8]. Through controlled drug release, prolonged circulation, enhanced tumor accumulation, and reduced exposure of healthy tissues, DDS may improve therapeutic efficacy while limiting adverse effects [9,10]. Various delivery platforms, including liposomes, micelles, and nanoparticles, have been developed for cancer treatment and are increasingly being investigated in lung cancer models [9]. To further enhance treatment specificity, both passive and active targeting strategies have been incorporated into DDS platforms. Passive targeting exploits the abnormal architecture and permeability of tumor vasculature, whereas active targeting utilizes ligands, antibodies, peptides, or other molecules that selectively recognize receptors expressed on cancer cells [9]. These approaches aim to increase drug accumulation within tumors and improve cellular uptake while reducing off-target toxicity. In addition to intravenous administration, alternative delivery routes such as inhalational, intratumoral, and intraperitoneal delivery have been explored to maximize local drug concentrations and improve therapeutic outcomes [11,12]. The composition of DDS, including polymeric, lipid-based, metallic, and protein-based carriers, plays an important role in determining drug loading capacity, release kinetics, biocompatibility, stability, and cellular internalization [13]. Among these platforms, polymeric nanoparticles have attracted considerable interest because of their versatility, biocompatibility, and ability to support controlled drug release and targeted delivery [10] A wide range of chemotherapeutic agents, including cisplatin, paclitaxel, docetaxel, and doxorubicin, have been successfully incorporated into DDS platforms for lung cancer treatment. Furthermore, combination chemotherapy approaches, such as cisplatin combined with paclitaxel or docetaxel, have demonstrated enhanced antitumor activity by targeting multiple biological pathways, overcoming resistance mechanisms, and improving overall treatment response [14]. Incorporation of these combinations into DDS platforms may further enhance therapeutic performance through coordinated drug delivery, optimized pharmacokinetics, and controlled release profiles [15]. Most DDS platforms are initially evaluated in vitro using lung cancer cell lines before progressing to animal models. Preclinical studies have reported encouraging results; however, therapeutic outcomes vary considerably depending on the delivery platform, targeting strategy, chemotherapeutic payload, route of administration, tumor model, and treatment schedule. Similar benefits of DDS-enhanced chemotherapy have been reported across multiple malignancies, including breast, liver, brain, colorectal, and lung cancers [13,14,15,16,17,18,19]. Although several reviews have summarized recent advances in nanocarrier-based therapies for lung cancer [9], a comprehensive quantitative evaluation of DDS efficacy across experimental lung cancer models remains lacking. Moreover, the relative contribution of key factors such as DDS type, targeting strategy, chemotherapeutic agent, route of administration, and combination therapy to treatment outcomes has not been systematically assessed. The novelty of the resent review lies in its quantitative and comparative focus. While recent reviews have mainly summarized nanocarrier-based strategies for lung cancer narratively, this study synthesizes controlled in vivo preclinical evidence and compares treatment effects across delivery platform, targeting strategy, chemotherapeutic payload, route of administration, and combination-treatment approach. Therefore, the aim of this systematic review and meta-analysis was to evaluate and compare the therapeutic efficacy of different DDS-based chemotherapeutic strategies in animal models of lung cancer and to identify the delivery-related factors associated with improved antitumor activity.

## 2. Materials and Methods

### 2.1. Search Strategy and Selection Criteria

This systematic review and meta-analysis was conducted in accordance with the Preferred Reporting Items for Systematic Reviews and Meta-Analyses (PRISMA 2020) guidelines [20]. The research question and eligibility criteria were defined using the PECOS framework (Population, Intervention, Comparison, Outcomes, and Study Design) and are presented in Table S1. As this study involved the analysis of previously published animal studies and did not include new experiments involving humans or animals, ethical approval and informed consent were not required. The review was not registered in PROSPERO because the database does not accept systematic reviews exclusively based on preclinical animal studies. A predefined internal protocol was developed before full-text screening and data extraction. The protocol specified the PECOS framework, databases, eligibly criteria, primary and secondary outcomes, data extraction, variables, risk-of-bias assessment and plane subgroup analyses. Selective reporting bias was mitigated by applying predefined eligibility criteria, duplicate independent screening, duplicate data extraction, consensus resolution with a third reviewer, and transparent reporting of all prespecified subgroup analyses. A comprehensive literature search was performed from database inception to 15 February 2025, using PubMed/MEDLINE, Embase, Scopus, Google Scholar, the Cochrane Central Register of Controlled Trials, and ClinicalTrials.gov. Google Scholar was used as a supplementary source because of its broad indexing, and screening was restricted to the first 200 records sorted by relevance using a predefined stopping rule. Additional searches included selected conference and organizational sources to identify potentially relevant gray literature. Unpublished animal studies and dissertations were not systematically included when quantitative tumor-volume data required for effect-size calculation were unavailable or insufficiently reported. The complete search strategy is provided in Table S2. No language restrictions were applied. Relevant search terms and filters were used to identify studies involving animal models of lung cancer treated with drug delivery system (DDS)-based chemotherapeutic interventions. Two reviewers independently screened titles and abstracts, assessed full-text articles for eligibility, and determined study inclusion. Any disagreements were resolved through discussion with a third reviewer. Data extraction was conducted independently by two reviewers using a predefined data extraction form. Information collected included study characteristics, tumor model, DDS platform, chemotherapeutic agent, targeting strategy, route of administration, treatment protocol, and outcome measures. Extracted data were cross-checked, and discrepancies were resolved by consensus.

The methodological quality of the included studies was independently assessed by two reviewers using the SYRCLE risk-of-bias tool and categorized as low, high, or unclear risk of bias [21]. Studies were considered eligible if they: (i) evaluated a chemotherapeutic agent delivered through a DDS in mouse models of lung cancer; (ii) included a comparator group receiving the corresponding free-drug or control treatment; (iii) reported sufficient quantitative data for effect-size calculation; and (iv) provided adequate information regarding DDS characteristics and treatment protocols. Studies not meeting these criteria were excluded. Immunotherapy combinations and immune-targeting nanocarriers were outside the predefined scope of this review, which focused on DDS-based chemotherapy. These approaches were not analyzed separately because they involve different mechanisms, immune-system dependence, model requirements and outcome expectations.

### 2.2. Outcome Variables

The primary outcome was tumor growth inhibition measured as tumor volume (mm^3^), because this was the most consistently reported quantitative endpoint across the included studies. Tumor weight was also extracted when reported. Secondary outcomes included survival and other indicators of treatment response when available. Toxicity, pharmacokinetic, biodistribution, metastasis, recurrence, and functional or quality-of-life surrogate outcomes were considered qualitatively where reported but were not quantitatively pooled because they were infrequently reported, inconsistently defined, measured at different time points, or presented using heterogeneous units and methods. Prespecified subgroup analyses were conducted according to DDS type, chemotherapeutic agent, targeting strategy, route of administration, and treatment approach.

### 2.3. Data Synthesis and Statistical Analyses

Meta-analyses were performed using Review Manager (RevMan) version 5.4.1 (The Cochrane Collaboration, London, UK). Statistical significance was defined as a two-tailed *p*-value < 0.05. Treatment effects for continuous outcomes were expressed as weighted mean differences (WMDs) with corresponding 95% confidence intervals (CIs) [22]. WMD was selected because the primary outcome was expressed as tumor volume in mm^3^, allowing the pooled effect to remain on a clinically and experimentally interpretable scale. Standardized mean difference was not used because it would have produced unitless estimates that are less directly interpretable for tumor volume reduction. Nevertheless, differences in baseline tumor size and outcome-assessment timing were acknowledged as contributors to heterogeneity. A random-effects model was applied to account for expected methodological and biological variability among studies. Statistical heterogeneity was assessed using Cochran’s Q test and quantified using the I^2^ statistic. Heterogeneity was interpreted as low (I^2^ < 25%), moderate (I^2^ = 25–50%), or high (I^2^ > 50%). Between-study variance was estimated using the τ^2^ statistic within the random-effects framework [23]. Cochran’s Q statistic was also reported for pooled analyses. Because substantial heterogeneity was expected, pooled estimates were interpreted as average effect across diverse experimental conditions rather than precise estimates for individual nanocarrier formulations. Prediction intervals were calculated for the main pooled analysis where sufficient data were available to indicate the expected range of effects in the future comparable study. Prespecified subgroup analyses were conducted to explore potential sources of heterogeneity, including targeting strategy, DDS platform, chemotherapeutic agent, route of administration, and treatment approach (single-agent versus combination therapy). The potential influence of study quality and risk of bias was considered during interpretation of the pooled findings. Publication bias was assessed by visual inspection of funnel plots when at least 10 studies or experiments were available. Egger’s regression and trim-and-fill analyses were not performed because several subgroups included fewer than 10 experiments, some studies contributed multiple experiments, and heterogeneity was substantial, which may make regression-based publication bias tests underpowered or misleading. Meta regression was considered but not performed because key covariates, including nanoparticle size, PDI, zeta potential, drug loading, ligand density, release kinetics, treatment duration, and tumor model category, were inconsistently reported and subgroup sample sizes were limited [24,25,26,27].

## 3. Results

### 3.1. Characteristics of Included Studies

The initial search identified 44,051 records, of which 644 were considered potentially relevant after title and abstract screening. Following full-text assessment, 30 studies comprising 47 experiments met the eligibility criteria and were included in the meta-analysis [28,29,30,31,32,33,34,35,36,37,38,39,40,41,42,43,44,45,46,47,48,49,50,51,52,53,54,55,56,57] (Figure S1).

The characteristics of the included studies are summarized in Table S3. Nanoparticles were the most frequently used DDS platform, appearing in 18 studies and 31 experiments [30,31,32,33,34,35,36,37,38,40,43,49,50,52,53,54,56,57]. Liposomal systems were evaluated in seven studies and 10 experiments [28,29,39,44,45,46,47], whereas micellar formulations were reported in five studies and six experiments [38,39,42,51,55]. Active targeting was used in 45% of the included studies, most commonly through hyaluronic acid, aptamers, or folic acid ligands [29,30,35,36,37,38,40,47,48,50,53,54,57], whereas passive targeting was used in 55% [28,31,32,33,34,39,41,42,43,44,45,46,49,51,52,55,56].

**Figure 1:**
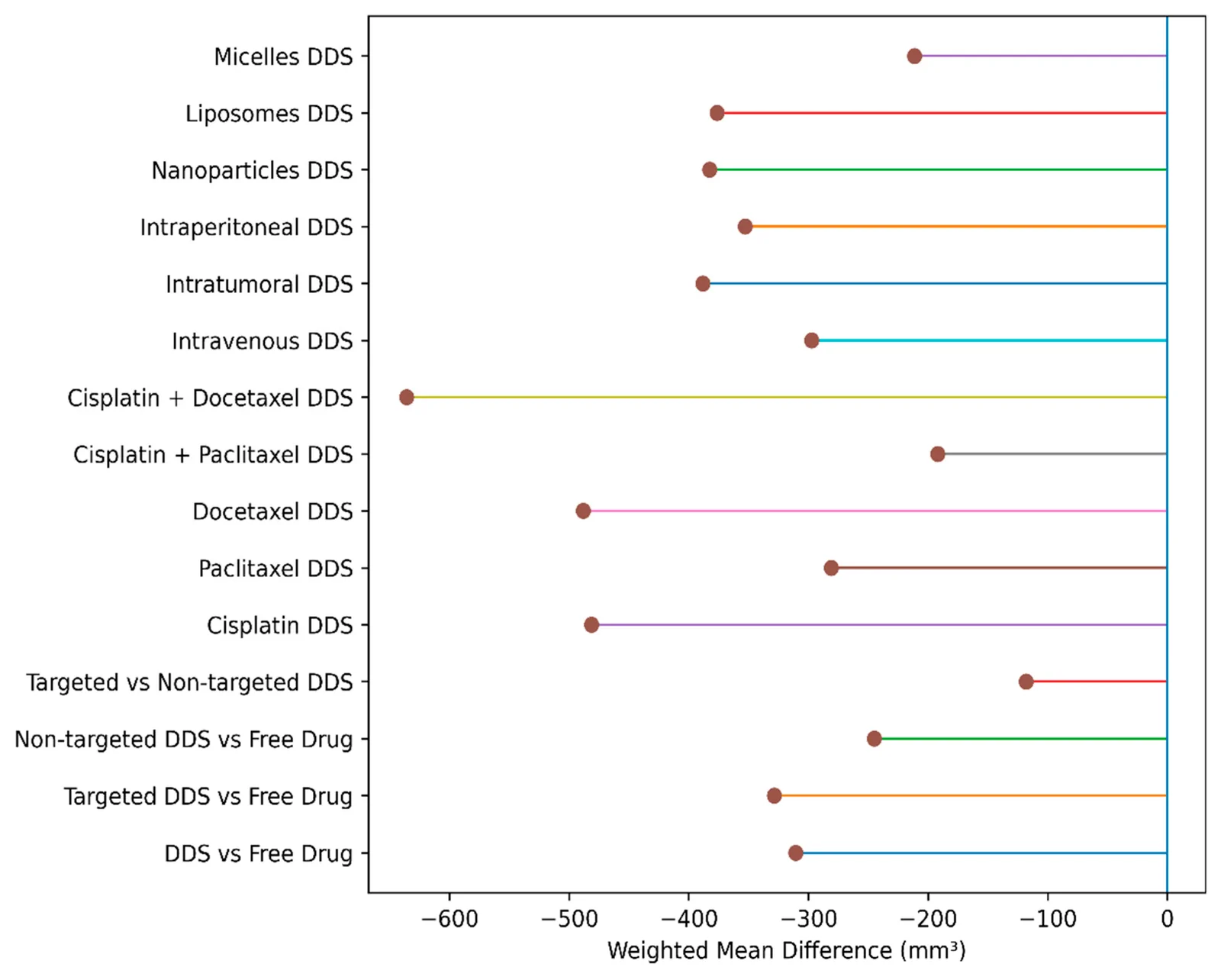
Summary of pooled effects of drug delivery systems (DDS) on tumor growth inhibition in experimental lung cancer models. The figure summarizes the weighted mean differences (WMD) in tumor volume reduction across the main subgroup analyses, including targeted and non-targeted DDS, different chemotherapeutic agents, and combination therapies.

Paclitaxel was the most frequently investigated chemotherapeutic agent, appearing in 12 studies with 17 experiments [29,30,34,35,39,40,42,44,49,51,52,54,55,56], followed by cisplatin in 12 studies with 14 experiments [28,30,31,33,41,42,44,46,48,50,53,57] and docetaxel in six studies with six experiments [32,33,34,36,37,45]. Combination regimens most commonly involved cisplatin plus paclitaxel, reported in four studies with six experiments [30,42,44,54], and cisplatin plus docetaxel, reported in one study with two experiments [47]. Intravenous administration was the predominant route, used in 24 studies and 37 experiments [28,29,30,31,32,33,34,35,36,40,41,42,43,44,45,47,48,49,50,51,52,53,54,55,57], whereas intraperitoneal delivery was reported in two studies [37,39] and intratumoral delivery in two studies [43,56]. Animal sex was reported as female in 14 studies and male in 11 studies, whereas five studies did not specify sex. Table 1 summarizes the pooled effect estimates across all outcomes and subgroup analyses, highlighting the overall and stratified efficacy of DDS-based chemotherapy in preclinical lung cancer models. Figure 1 graphically presents these findings, facilitating comparison of effect sizes across different DDS platforms, targeting strategies, and treatment regimens. Most included experiments used subcutaneous xenograft models, whereas orthotopic, syngeneic, metastatic, and genetically engineered mouse models were absent or too infrequently represented for meaningful quantitative subgroup comparisons. Therefore, tumor model category was considered during interpretation but was not analyzed as a separate pooled subgroup.

### 3.2. Overall Effect of DDS-Based Chemotherapy on Tumor Growth Inhibition

Across the included studies, treatment with chemotherapeutic agents encapsulated in drug delivery systems (DDS) resulted in significantly greater tumor growth inhibition compared with administration of the corresponding free drugs in experimental lung cancer models. The pooled meta-analysis demonstrated a weighted mean difference (WMD) in tumor volume of −310.67 (95% CI: −375.51 to −245.83; *p* < 0.001), indicating a statistically significant reduction in tumor size in animals treated with DDS-based formulations. Substantial heterogeneity was present; therefore, this pooled estimate should be interpreted as the average effect across heterogeneous preclinical experiments rather than as a precise prediction for any individual DDS formulation. The prediction interval for the main analysis was wide, indicating that the magnitude of the effect may vary considerably in the future comparable studies. The overall effect estimate is presented in Figure S2.

### 3.3. Efficacy of Targeted and Non-Targeted DDS Versus Free Drug

Subgroup analysis comparing targeted and non-targeted DDS formulations with free-drug treatments showed that both strategies were associated with significant tumor growth inhibition. Targeted DDS formulations demonstrated a pooled WMD of –328.64, whereas non-targeted DDS formulations showed a pooled WMD of –244.91. Both subgroup effects were statistically significant (*p* < 0.001). The results of this subgroup analysis are illustrated in Figure S3.

### 3.4. Comparative Efficacy of Targeted Versus Non-Targeted DDS

A direct comparison between targeted and non-targeted DDS formulations demonstrated that targeted DDS achieved a significantly greater reduction in tumor volume. The pooled weighted mean difference between the two groups was −118.13 (95% CI: −164.28 to −71.98; *p* < 0.001). These results are presented in Figure S4.

### 3.5. Efficacy According to Chemotherapeutic Agent Delivered via DDS

Subgroup analysis based on the chemotherapeutic agents encapsulated within DDS revealed differences in tumor growth inhibition across drug classes. Docetaxel-based DDS demonstrated the largest reduction in tumor volume (WMD: −488.12), followed by cisplatin-based DDS (WMD: −481.20) and paclitaxel-based DDS (WMD: −281.05). All pooled estimates indicated statistically significant tumor growth inhibition compared with free-drug treatments (*p* < 0.001). Because the docetaxel subgroup comprised fewer experiments than cisplatin and paclitaxel subgroups, these results should be interpreted with caution. The results of these subgroup analyses are presented in Figure S5.

### 3.6. Efficacy of Combination Chemotherapy Delivered via DDS

Subgroup analysis evaluating combination chemotherapy delivered via DDS showed significantly greater tumor growth inhibition compared with the corresponding free-drug combinations. The combination of cisplatin and paclitaxel encapsulated in DDS demonstrated a pooled WMD of −191.93 (*p* < 0.001). The combination of cisplatin and docetaxel delivered via DDS showed a larger pooled effect, with a WMD of −635.82 (*p* < 0.001). However, this subgroup was based on only two experiments and should therefore be interpreted with caution. These findings are presented in Figure S6.

### 3.7. Efficacy According to Route of Administration

Subgroup analysis based on the route of administration demonstrated variation in treatment effects across delivery methods. Intratumoral administration of DDS formulations resulted in a pooled WMD of −388.29 (*p* < 0.001). Intraperitoneal administration yielded a pooled WMD of −352.82, which did not reach statistical significance (*p* = 0.19). Intravenous administration demonstrated a statistically significant reduction in tumor volume with a pooled WMD of −297.30 (*p* < 0.001). The results are illustrated in Figure S7.

### 3.8. Efficacy According to DDS Platform

Subgroup analysis based on the type of drug delivery system demonstrated that all DDS platforms were associated with significant tumor growth inhibition compared with free-drug treatments. Nanoparticle-based systems showed the largest pooled effect (WMD: −382.71; *p* < 0.001), followed by liposomal systems (WMD: −376.33; *p* < 0.001) and micellar systems (WMD: −211.45; *p* < 0.001). However, no formal statistical compression was performed between DDS platform categories; therefore, these differences should be regarded as descriptive rather than comparative. These findings are summarized in Figure S8.

### 3.9. Risk-of-Bias Assessment

**Figure 2:**
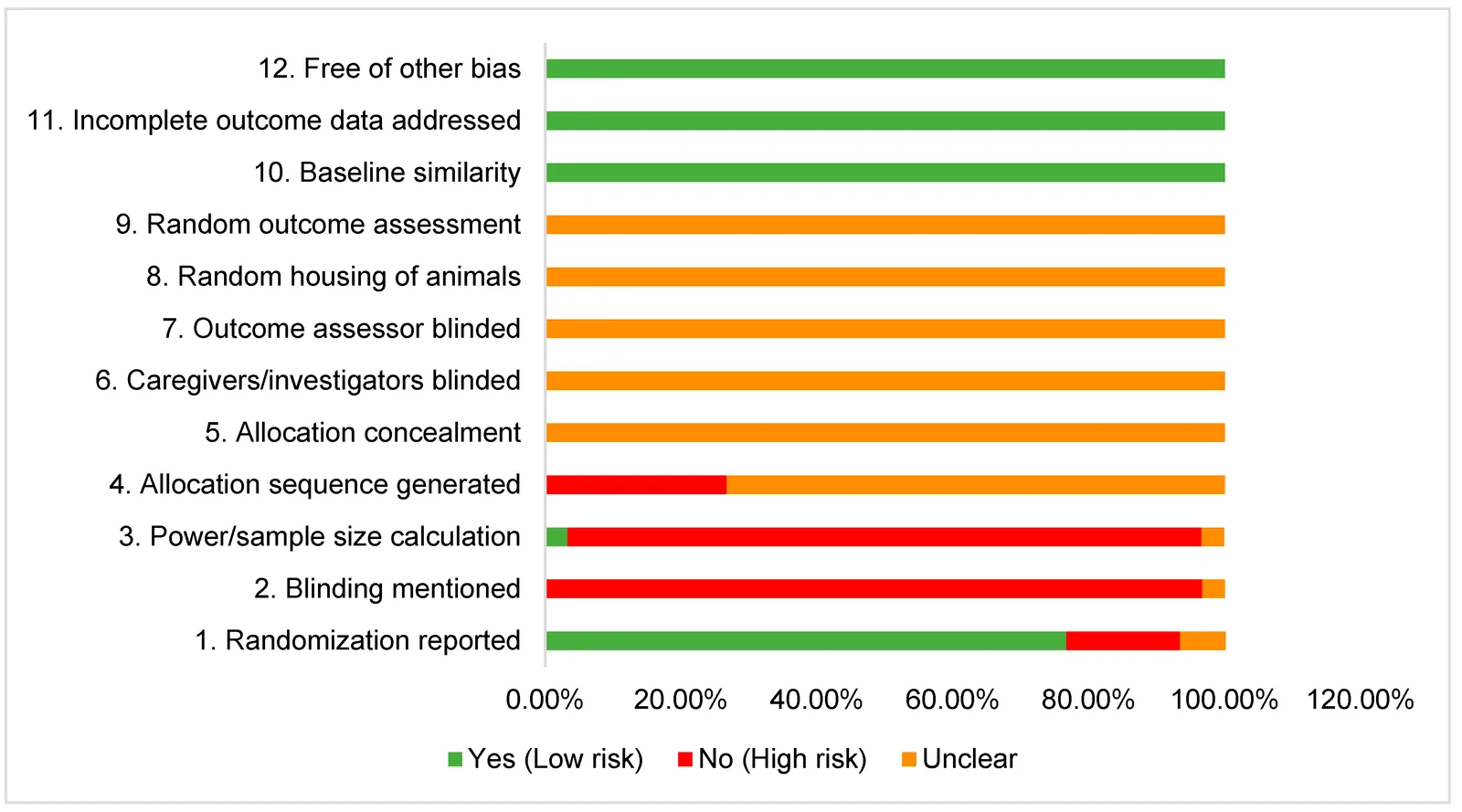
Risk-of-bias assessment of the included studies.

As summarized in Table S4 and Figure 2, the methodological reporting of the included studies showed several limitations. Randomization procedures were reported in a proportion of the studies, whereas other important methodological safeguards, including allocation concealment, blinding of caregivers or investigators, and blinding of outcome assessors, were rarely described. Similarly, information regarding random housing of animals and random selection for outcome assessment was generally not reported. Consequently, several domains of the SYRCLE risk-of-bias tool were classified as presenting an unclear risk of bias, primarily due to insufficient reporting of experimental procedures in the original studies. Only a small number of studies reported sample size or power calculations. In contrast, baseline comparability between experimental groups and the handling of incomplete outcome data were consistently reported across the included studies, and no additional sources of bias were identified. Due to incomplete reporting of methodological safeguards in several domains, many items were classified as unclear risk of bias rather than high risk, reflecting uncertainty related to reporting rather than confirmed methodological flaws. Therefore, unclear risk reflects incomplete reporting rather than confirmed methodological failure.

Above is the traffic light plot of the risk-of-bias assessment for the included preclinical animal studies using the SYRCLE risk-of-bias tool. Each column represents an individual study (*n* = 30), and each row corresponds to a specific methodological domain evaluated in the risk-of-bias assessment. Green circles indicate low risk of bias, yellow circles indicate unclear risk of bias, and red circles indicate high risk of bias. The assessment shows that although some studies reported randomization procedures, most methodological safeguards such as allocation concealment, blinding of investigators, and blinding of outcome assessors were insufficiently reported, resulting in several domains being classified as unclear risk of bias. A strict sensitivity analysis excluding all studies with any unclear risk-of-bias domain was not feasible, because most animal studies lacked complete reporting for at least one SYRCLE domain. Excluding all such studies would have substantially reduced the dataset and may have introduced selection bias on reporting quality. Therefore, the pooled findings were interpreted in the context of the overall risk-of-bias profile.

## 4. Discussion

This systematic review and meta-analysis provides quantitative evidence that drug delivery system (DDS)-based chemotherapy improves tumor growth inhibition compared with conventional free-drug treatment in experimental lung cancer models. Across a broad range of murine models, chemotherapeutic agents, and delivery platforms, DDS-based formulations consistently reduced tumor volume, supporting the hypothesis that optimized drug delivery can enhance the therapeutic performance of anticancer agents. The observed benefit of DDS-based therapies is biologically plausible and aligns with current understanding of the barriers limiting conventional chemotherapy in lung cancer. Systemically administered anticancer drugs often exhibit rapid distribution to healthy tissues, dose-limiting toxicity, inadequate tumor penetration, and the development of treatment resistance. DDS platforms are designed to address these challenges by improving drug stability, prolonging circulation time, enhancing tumor accumulation, and enabling controlled drug release. The overall reduction in tumor burden observed in this analysis suggests that these advantages translate into measurable improvements in therapeutic efficacy in preclinical settings [9]. Targeted DDS formulations demonstrated greater antitumor activity than non-targeted systems. This finding is consistent with previous reports showing that ligand-mediated targeting can enhance selective uptake by tumor cells and improve intratumoral drug exposure [9]. However, the superior performance of targeted systems is unlikely to be explained solely by receptor recognition. Factors such as carrier composition, particle size, surface charge, colloidal stability, and drug-release kinetics may also contribute to improved treatment outcomes. Therefore, the efficacy of targeted DDS should be viewed as the result of multiple interacting formulation characteristics rather than a single design feature. Among the evaluated delivery platforms, nanoparticle-based systems produced the largest pooled reductions in tumor volume. Nanoparticles offer several advantages, including high drug-loading capacity, protection of unstable compounds, prolonged circulation, and the possibility of incorporating active targeting strategies. Similar observations have been reported in studies of lung and other solid tumors, where nanoparticle-based formulations improved drug accumulation within tumors and enhanced therapeutic responses [9]. The current findings suggest that these advantages may also be relevant in lung cancer and support continued development of nanoparticle-based approaches for pulmonary malignancies. Differences among DDS platforms may reflect multiple formulation-dependent mechanisms. Nanoparticles may provide flexible control over particle size, surface modification, drug loading, release kinetics, and active targeting. Liposomal systems may improve circulation time and encapsulation of selected agents, whereas micelles may improve solubilization of hydrophobic drugs but can be limited by colloidal stability and premature drug release. Additional factors, including surface charge, protein corona formation, cellular uptake, endosomal escape, tumor penetration, and route-dependent biodistribution, may also influence antitumor performance. These mechanistic differences further support interpreting subgroup rankings cautiously, because multiple formulation characteristics rather than platform type alone may contribute to the observed treatment effect. Accordingly, differences among DDS platforms should not be interpreted as evidence that one delivery platform is intrinsically superior to another because multiple formulation characteristics and study level factors may contribute to the observed subgroup effects. Therapeutic efficacy also varied according to the chemotherapeutic payload. DDS formulations containing docetaxel and cisplatin demonstrated larger pooled effects than paclitaxel-based systems. Although these differences should be interpreted cautiously, they may reflect variations in drug potency, pharmacokinetic behavior, tissue penetration, and sensitivity to controlled-release strategies. Furthermore, DDS-mediated combination therapies achieved greater tumor inhibition than corresponding free-drug combinations. However, this finding should not be interpreted as confirmed synergy unless supported by formal interaction testing in the original studies. An additive effect occurs when the combined effect of two agents approximates the expected sum of their individual effects, whereas synergy implies an effect greater than the expected additive effect and requires appropriate factorial or interaction-based assessment. Therefore, the present review uses cautious terminology such as combination benefit or enhanced tumor inhibition. Simultaneous delivery of multiple agents through a single carrier may improve drug exposure ratios at the tumor site, reduce pharmacokinetic mismatches, and enhance synergistic interactions between therapeutic agents. Similar benefits of co-delivery systems have been highlighted in recent reviews focusing on non-small cell lung cancer [58], where synchronized delivery of multiple therapeutic agents was associated with improved efficacy and the potential to overcome treatment resistance. These findings are in agreement with previous reports indicating that the success of combination therapy depends not only on the selection of therapeutic agents but also on their delivery strategy, administration schedule, and pharmacokinetic compatibility, all of which may influence treatment efficacy and therapeutic synergy in lung cancer [18]. Despite the overall positive findings, substantial heterogeneity was observed across studies. Such variability is expected in preclinical nanomedicine research because experimental designs differ considerably with respect to tumor models, cell lines, implantation methods, treatment schedules, dosing regimens, and nanocarrier composition. Consequently, the pooled estimates should be interpreted as average effects across diverse experimental conditions rather than precise predictions of efficacy for any individual DDS platform. This variability also highlights the complexity of translating preclinical findings into clinical applications. Clinically established nanocarrier systems, including liposomal formulation and albumin-bound paclitaxel, demonstrate that nanomedicine can reach clinical use when formulation, pharmacokinetics, safety, manufacturing, and reproducibility are sufficiently optimized. However, many experimental nanomedicines fail clinically despite promising animal efficacy. Potential reasons include limited replication in robust animal models, differences between murine and human tumor microenvironments, variable tumor vascular permeability, immune-system effects, off-target biodistribution, toxicity, scale-up and manufacturing challenges, insufficient batch-to-batch reproducibility, and incomplete characterization of critical quality attributes. The risk-of-bias assessment identified important weaknesses in methodological reporting. These findings emphasize that future preclinical studies should combine rigorous experimental design with standardized characterization of nanocarrier system to improve reproducibility and facilitate clinical translation. Information regarding randomization, allocation concealment, blinding, and sample size calculation was frequently missing or insufficiently described. These limitations are common in preclinical oncology research and may contribute to overestimation of treatment effects and reduced reproducibility. Improved adherence to reporting frameworks such as the ARRIVE guidelines would strengthen the reliability, transparency, and comparability of future animal studies evaluating DDS-based therapies.

### 4.1. Strengths and Limitations

The present study has several strengths. To our knowledge, it represents one of the first quantitative syntheses of preclinical evidence evaluating DDS-based chemotherapy specifically in lung cancer models. The inclusion of subgroup analyses enabled the assessment of factors that may influence treatment efficacy, including targeting strategy, delivery platform, chemotherapeutic payload, and route of administration, thereby allowing exploration of potential sources of between-study heterogeneity. In addition, methodological quality was systematically evaluated using the SYRCLE risk-of-bias tool. Several limitations should also be acknowledged. Considerable heterogeneity existed among the included studies, reflecting differences in experimental design, tumor models, treatment protocols, outcome timing, dosing regimen and DDS characteristics. Therefore, the overall pooled estimate should therefore be interpreted as representing the average effect of DDS -based chemotherapy across heterogeneous preclinical settings rather than implying comparable efficacy across individual nanocarrier platforms. Meta regression could not be performed because important formulation characteristics and experimental covariates were inconsistently reported across studies; the influence of these variables could not be examined quantitatively and therefore remains uncertain. Important outcomes including toxicity, pharmacokinetic, biodistribution, survival, metastasis, recurrence, and functional endpoints could not be quantitatively synthesized because of inconsistent definitions and incomplete reporting across the studies. Many studies lacked detailed reporting of methodological procedures, limiting the assessment of internal validity. Furthermore, the analysis was based on aggregate published data and did not include individual animal-level data. Publication bias assessment was limited, and regression-based tests were not considered reliable for small and heterogeneous subgroups. Finally, most studies used murine subcutaneous models, which do not fully reproduce the biological complexity, tumor microenvironment–immune interactions, metastatic behavior, and treatment response observed in human lung cancer.

### 4.2. Implications for Practice and Future Research

The findings of this study support the continued development of DDS-based chemotherapy as a strategy to improve anticancer drug delivery in lung cancer. Targeted DDS formulations, nanoparticle-based platforms, and combination-treatment approaches appear particularly promising and warrant further investigation [9,58]. Future preclinical DDS studies should report nanocarrier characterization in a standardized manner, as well as particle size, size distribution, PDI, zeta potential, morphology, drug loading, encapsulation efficiency, in vitro and in vivo release kinetics, stability in biological media, ligand density, protein corona behavior, sterility, endotoxin status, and batch reproducibility. They should also include standardized toxicity assessment, pharmacokinetics, biodistribution, toxicity, and long-term treatment outcomes. Also, preclinical studies should routinely report survival, metastatic burden, recurrence and functional outcomes in addition to tumor volume. Particular attention should also be given to dosing regimens, treatment scheduling, and delivery technologies, as these factors may substantially influence the effectiveness of combination therapies and their translational potential [58,59]. Greater consistency in reporting nanocarrier characteristics and animal-study methodology would facilitate comparisons across studies and improve the quality of future evidence. Ultimately, well-designed preclinical investigations and carefully planned translational studies will be essential to identify the DDS platforms with the greatest potential for clinical application in lung cancer treatment [59].

## 5. Conclusions

This systematic review and meta-analysis indicates that drug delivery system (DDS)-based chemotherapy is associated with greater tumor growth inhibition than corresponding free-drug treatment in preclinical lung cancer models. Larger average effects were observed in several subgroup analyses, including targeted DDS, nanoparticle-based systems, and selected combination or docetaxel-based formulations; however, these findings should be interpreted cautiously because of heterogeneity, limited subgroup sizes, and potential confounding by formulation and study-design characteristics. The results support continued translational development of nanocarrier-based chemotherapy, but they do not establish clinical efficacy. Future studies require standardized reporting, rigorous experimental design, and comprehensive evaluation of pharmacokinetics, biodistribution, toxicity, and clinically relevant outcomes to improve reproducibility and translational relevance.

## Figures and Tables

**Table 1:** Summary of pooled treatment effects from the meta-analysis.

Outcome/Subgroup	k	Animals DDS	Animals Control	WMD	95% CI	Z	*p* Overall	Cochran’s Q (χ^2^)	df	p Heterogeneity	I^2^ (%)	τ^2^
Overall DDS vs. free drug	47	486	318	−310.67	−375.51 to −245.83	9.39	<0.00001	169.02	46	<0.00001	73	29,514.61
Targeted DDS vs. free drug	23	154	146	−328.64	−416.05 to −241.23	7.37	<0.00001	66.59	22	<0.00001	67	24,183.99
Non-targeted DDS vs. free drug	32	214	213	−244.91	−317.72 to −172.10	6.59	<0.00001	113.26	31	<0.00001	73	25,008.02
Targeted vs. non-targeted DDS	17	110	106	−118.13	−164.28 to −71.98	5.02	<0.00001	36.23	16	0.003	56	3866.62
Cisplatin DDS vs. free drug	14	93	91	−481.20	−691.12 to −271.28	4.49	<0.00001	127.40	13	<0.00001	90	132,857.48
Paclitaxel DDS vs. free drug	17	142	107	−281.05	−385.11 to −176.99	5.29	<0.00001	47.75	16	<0.0001	66	25,194.72
Docetaxel DDS vs. free drug	6	47	41	−488.12	−817.96 to −158.27	2.90	0.004	36.34	5	<0.00001	86	137,629.42
Combination: cisplatin + paclitaxel	6	62	38	−191.93	−292.53 to −91.33	3.74	0.0002	16.82	5	0.005	70	10,296.10
Combination: cisplatin + docetaxel	2	24	12	−635.82	−963.43 to −308.21	3.80	0.0001	0.04	1	0.84	0	0.00
Intraperitoneal administration	2	32	16	−352.82	−879.32 to 173.68	1.31	0.19	4.27	1	0.04	77	111,618.53
Intravenous administration	37	313	241	−297.30	−364.50 to −230.10	8.67	<0.00001	114.33	36	<0.00001	69	22,983.28
Intratumoral administration	2	11	10	−388.29	−896.10 to 119.52	1.50	0.13	8.10	1	0.004	88	118,509.04
Nanoparticle DDS	31	301	198	−382.71	−483.70 to −281.73	7.43	<0.00001	187.01	30	<0.00001	84	57,155.11
Liposome DDS	10	198	65	−376.33	−547.61 to −205.04	4.31	<0.00001	31.14	9	0.0003	71	47,171.32
Micelle DDS	6	46	41	−211.45	−337.99 to −84.92	3.28	0.001	12.22	5	0.03	59	13,733.43

## Data Availability

The data supporting the findings of this study are available from the corresponding author upon reasonable request. The extracted data used in the meta-analysis were obtained from previously published studies cited in this article.

## Supplementary Materials

The following supporting information can be downloaded at https://www.mdpi.com/article/10.3390/arm94040050/s1.

## Abbreviations

The following abbreviations are used in this manuscript:

DDS	Drug Delivery System
IV	Intravenous
IP	Intraperitoneal
IT	Intratumoral
NP	Nanoparticle
CDDP	Cisplatin
PTX	Paclitaxel
DTX	Docetaxel
DOX	Doxorubicin
FA	Folic Acid
APT	Aptamer

## References

[1] Smolarz B., Łukasiewicz H., Samulak D., Piekarska E., Kołaciński R., Romanowicz H. (2025). Lung cancer: Epidemiology, pathogenesis, treatment and molecular aspect. Int. J. Mol. Sci..

[2] Sumiya R., Matsunaga T., Suzuki K. (2025). Lung cancer in young individuals; risk factors and epidemiology. J. Thorac. Dis..

[3] Neupane B.K., Acharya B., Cao C., Xu M., Bhattarai H., Yang Y., Wang S. (2024). A systematic review of spatial and temporal epidemiological approaches focusing on lung cancer risk associated with particulate matter. BMC Public Health.

[4] Li Y., Xiao X., Han Y., Cheng C., Fernandes G.F., Slewitzke S.E., Rosenberg S.M., Zhu M., Byun J., Bossé Y. (2024). Lung cancer in ever- and never-smokers: Findings from multi-population GWAS studies. Cancer Epidemiol. Biomark. Prev..

[5] Riely G.J., Wood D.E., Aisner D.L., Loo B.W., Axtell A.L., Bauman J.R., Bharat A., Chang J.Y., Desai A., Dilling T.J. (2025). NCCN Guidelines^®^ Insights: Non-Small Cell Lung Cancer, Version 7.2025. J. Natl. Compr. Cancer Netw..

[6] Siegel R.L., Miller K.D., Wagle N.S., Jemal A. (2024). Cancer statistics, 2024. CA Cancer J. Clin..

[7] Su W.C., Lai W.W., Chen H.H., Hsiue T.R., Chen C.W., Huang W.T., Chen T.Y., Tsao C.J., Wang N.S. (2003). Combined intrapleural and intravenous chemotherapy, and pulmonary irradiation, for treatment of patients with lung cancer presenting with malignant pleural effusion. A pilot study. Oncology.

[8] Koliqi R., Breznica P., Daka A., Koshi B. (2021). Application of Design of Expert software for evaluating the influence of formulation variables on the encapsulation efficacy, drug content and particle size of PEO-PPO-PEO/Poly (DL-lactide-co-caprolactone) nanoparticles as carriers for SN-38. Med. Pharm. Rep..

[9] Li Z., Liu C., Di Z., Zhang G., You S., Chao S., Ren Y. (2026). Recent Advances in Nanocarrier-Based Drug Delivery Systems for Lung Cancer. Int. J. Nanomed..

[10] Koliqi R., Uskoković V., Selmani P.B., Grapci A.D., Mahapatra C. (2025). Polymeric nanoparticles for wound healing. Pharm. Nanotechnol..

[11] Shi J., Kantoff P.W., Wooster R., Farokhzad O.C. (2017). Cancer nanomedicine: Progress, challenges and opportunities. Nat. Rev. Cancer.

[12] Ara N., Hafeez A. (2024). Nanocarrier-mediated drug delivery via inhalational route for lung cancer therapy: A systematic and updated review. AAPS PharmSciTech.

[13] Zhang S., Li R., Jiang T., Gao Y., Zhong K., Cheng H., Chen X., Li S. (2024). Inhalable nanomedicine for lung cancer treatment. Smart Mater. Med..

[14] Zeinali R., Zaeifi D., Zolfaghari-Moghaddam S.Y., Paul M.K., Biazar E. (2025). Current Advances in Nanocarriers for Cancer Therapy. Int. J. Nanomed..

[15] Chen D., Liu X., Lu X., Tian J. (2023). Nanoparticle drug delivery systems for synergistic delivery of tumor therapy. Front. Pharmacol..

[16] Deng Z., Siraj A.A., Lowry I., Ruan E., Patel R., Gao W., Kalin T.V., Kalinichenko V.V. (2025). Nanoparticle-based delivery systems for synergistic therapy in lung cancers. Bioengineering.

[17] Peer D., Karp J.M., Hong S., Farokhzad O.C., Margalit R., Langer R. (2020). Nanocarriers as an emerging platform for cancer therapy. Nat. Nanotechnol..

[18] Wu J., Bao Q., Wang X., Chen H., Chen X., Wen Y., Chen J. (2025). Research progress of co-delivery nanoparticle drug delivery systems in non-small cell lung cancer: A review. Colloids Surf. B Biointerfaces.

[19] Anselmo A.C., Mitragotri S. (2021). Nanoparticles in the clinic: An update. Bioeng. Transl. Med..

[20] Blanco E., Shen H., Ferrari M. (2015). Principles of nanoparticle design for overcoming biological barriers to drug delivery. Nat. Biotechnol..

[21] Feng Q., Liu Y., Huang J., Chen K., Huang J. (2022). Nanotechnology-based drug delivery systems for brain cancer treatment: Recent progress and future prospects. J. Control. Release.

[22] Zhao Y., Hong X., Chan H., Wong Y.S., Huang Y. (2023). Current advances in nanotechnology for colorectal cancer diagnosis and treatment. J. Nanobiotechnol..

[23] Page M.J., McKenzie J.E., Bossuyt P.M., Boutron I., Hoffmann T.C., Mulrow C.D., Shamseer L., Tetzlaff J.M., Akl E.A., Brennan S.E. (2021). The PRISMA 2020 statement: An updated guideline for reporting systematic reviews. BMJ.

[24] Hooijmans C.R., Rovers M.M., de Vries R.B., Leenaars M., Ritskes-Hoitinga M., Langendam M.W. (2014). SYRCLE’s risk of bias tool for animal studies. BMC Med. Res. Methodol..

[25] Hozo S.P., Djulbegovic B., Hozo I. (2005). Estimating the mean and variance from the median, range, and the size of a sample. BMC Med. Res. Methodol..

[26] Higgins J.P.T., Thompson S.G. (2002). Quantifying heterogeneity in a meta-analysis. Stat. Med..

[27] Egger M., Davey Smith G., Schneider M., Minder C. (1997). Bias in meta-analysis detected by a simple, graphical test. BMJ.

[28] Cao Z.T., Chen Z.Y., Sun C.Y., Li H.J., Wang H.X., Cheng Q.Q., Zuo Z.Q., Wang J.L., Liu Y.Z., Wang Y.C. (2016). Overcoming tumor resistance to cisplatin by cationic lipid-assisted prodrug nanoparticles. Biomaterials.

[29] Guo S., Zhang Y., Wu Z., Zhang L., He D., Li X., Wang Z. (2019). Synergistic combination therapy of lung cancer: Cetuximab functionalized nanostructured lipid carriers for the co-delivery of paclitaxel and 5-Demethylnobiletin. Biomed. Pharmacother..

[30] He Z., Huang J., Xu Y., Zhang X., Teng Y., Huang C., Wu Y., Zhang X., Zhang H., Sun W. (2015). Co-delivery of cisplatin and paclitaxel by folic acid conjugated amphiphilic PEG-PLGA copolymer nanoparticles for the treatment of non-small lung cancer. Oncotarget.

[31] Iyer R., Nguyen T., Padanilam D., Xu C., Saha D., Nguyen K.T., Hong Y. (2020). Glutathione-responsive biodegradable polyurethane nanoparticles for lung cancer treatment. J. Control. Release.

[32] Jin G., Jin M., Jin Z., Gao Z., Yin X. (2016). Docetaxel-loaded PEG-albumin nanoparticles with improved antitumor efficiency against non-small cell lung cancer. Oncol. Rep..

[33] Jin Y., Wang Y., Liu X., Zhou J., Wang X., Feng H., Liu H. (2020). Synergistic combination chemotherapy of lung cancer: Cisplatin and doxorubicin conjugated prodrug loaded, glutathione and pH sensitive nanocarriers. Drug Des. Dev. Ther..

[34] Jung J., Park S.J., Chung H.K., Kang H.W., Lee S.W., Seo M.H., Park H.J., Song S.Y., Jeong S.Y., Choi E.K. (2012). Polymeric nanoparticles containing taxanes enhance chemoradiotherapeutic efficacy in non-small cell lung cancer. Int. J. Radiat. Oncol. Biol. Phys..

[35] Li S., Gray B.P., McGuire M.J., Brown K.C. (2011). Synthesis and biological evaluation of a peptide–paclitaxel conjugate which targets the integrin αvβ6. Bioorganic Med. Chem..

[36] Li T., Hu Z., Wang C., Yang J., Zeng C., Fan R., Guo J. (2020). PD-L1-targeted microbubbles loaded with docetaxel produce a synergistic effect for the treatment of lung cancer under ultrasound irradiation. Biomater. Sci..

[37] Liang Z., Yang N., Jiang Y., Hou C., Zheng J., Shi J., Zhang R., Li D., Liu Y., Zuo P. (2015). Targeting docetaxel-PLA nanoparticles simultaneously inhibit tumor growth and liver metastases of small cell lung cancer. Int. J. Pharm..

[38] Long J.T., Cheang T.Y., Zhuo S.Y., Zeng R.F., Dai Q.S., Li H.P., Fang S. (2014). Anticancer drug-loaded multifunctional nanoparticles to enhance the chemotherapeutic efficacy in lung cancer metastasis. J. Nanobiotechnol..

[39] Nguyen D.M., Lorang D., Chen G.A., Stewart I.V.J.H., Tabibi E., Schrump D.S. (2001). Enhancement of paclitaxel-mediated cytotoxicity in lung cancer cells by 17-allylamino geldanamycin: In vitro and in vivo analysis. Ann. Thorac. Surg..

[40] Shi H., Chen L., Liu Y., Wen Q., Lin S., Wen Q., Lu Y., Dai J., Li J., Xiao S. (2023). Bacteria-driven tumor microenvironment-sensitive nanoparticles targeting hypoxic regions enhances the chemotherapy outcome of lung cancer. Int. J. Nanomed..

[41] Wang S., Gou J., Wang Y., Tan X., Zhao L., Jin X., Tang X. (2021). Synergistic antitumor efficacy mediated by liposomal co-delivery of polymeric micelles of vinorelbine and cisplatin in non-small cell lung cancer. Int. J. Nanomed..

[42] Song W., Tang Z., Li M., Lv S., Sun H., Deng M., Liu H., Chen X. (2014). Polypeptide-based combination of paclitaxel and cisplatin for enhanced chemotherapy efficacy and reduced side-effects. Acta Biomater..

[43] Tang B.C., Fu J., Watkins D.N., Hanes J. (2010). Enhanced efficacy of local etoposide delivery by poly (ether-anhydride) particles against small cell lung cancer in vivo. Biomaterials.

[44] Wang G., Wang Z., Li C., Duan G., Wang K., Li Q., Tao T. (2018). RGD peptide-modified, paclitaxel prodrug-based, dual-drugs loaded, and redox-sensitive lipid-polymer nanoparticles for the enhanced lung cancer therapy. Biomed. Pharmacother..

[45] Wang Y., Guo M., Lin D., Liang D., Zhao L., Zhao R., Wang Y. (2021). Docetaxel-loaded exosomes for targeting non-small cell lung cancer: Preparation and evaluation in vitro and in vivo. Drug Deliv..

[46] Wang X., Liu G., Pu X., Ren T., Zhang F., Shen M., Zhu Y., Kros A., Yang J. (2025). Combating cisplatin-resistant lung cancer using a coiled-coil lipopeptides modified membrane fused drug delivery system. J. Control. Release.

[47] Wu R., Zhang Z., Wang B., Chen G., Zhang Y., Deng H., Tang Z., Mao J., Wang L. (2020). Combination chemotherapy of lung cancer–co-delivery of docetaxel prodrug and cisplatin using aptamer-decorated lipid–polymer hybrid nanoparticles. Drug Des. Dev. Ther..

[48] Xiong Y., Zhao Y., Miao L., Lin C.M., Huang L. (2016). Co-delivery of polymeric metformin and cisplatin by self-assembled core-membrane nanoparticles to treat non-small cell lung cancer. J. Control. Release.

[49] Yang D., Liu X., Jiang X., Liu Y., Ying W., Wang H., Bai H., Taylor W.D., Wang Y., Clamme J.P. (2012). Effect of molecular weight of PGG–paclitaxel conjugates on in vitro and in vivo efficacy. J. Control. Release.

[50] Yang S.J., Pai J.A., Shieh M.J., Chen J.L., Chen K.C. (2023). Cisplatin-loaded gold nanoshells mediate chemo-photothermal therapy against primary and distal lung cancers growth. Biomed. Pharmacother..

[51] Yuan Z.Q., Li J.Z., Liu Y., Chen W.L., Yang S.D., Zhang C.G., Zhu W.J., Zhou X.F., Liu C., Zhang X.N. (2015). Systemic delivery of micelles loading with paclitaxel using N-succinyl-palmitoyl-chitosan decorated with cRGDyK peptide to inhibit non-small-cell lung cancer. Int. J. Pharm..

[52] Yuan X., Ji W., Chen S., Bao Y., Tan S., Lu S., Wu K., Chu Q. (2016). A novel paclitaxel-loaded poly (d, l-lactide-co-glycolide)-Tween 80 copolymer nanoparticle overcoming multidrug resistance for lung cancer treatment. Int. J. Nanomed..

[53] Bi Y.Y., Chen Q., Yang M.Y., Xing L., Jiang H.L. (2024). Nanoparticles targeting mutant p53 overcome chemoresistance and tumor recurrence in non-small cell lung cancer. Nat. Commun..

[54] He Z., Shi Z., Sun W., Ma J., Xia J., Zhang X., Chen W., Huang J. (2016). Hemocompatibility of folic-acid-conjugated amphiphilic PEG-PLGA copolymer nanoparticles for co-delivery of cisplatin and paclitaxel: Treatment effects for non-small-cell lung cancer. Tumor Biol..

[55] Zhang L., Liu Z., Kong C., Liu C., Yang K., Chen H., Huang J., Qian F. (2018). Improving drug delivery of micellar paclitaxel against non-small cell lung cancer by coloading itraconazole as a micelle stabilizer and a tumor vascular manipulator. Small.

[56] Zhao T., Chen H., Dong Y., Zhang J., Huang H., Zhu J., Zhang W. (2013). Paclitaxel-loaded poly (glycolide-co-ε-caprolactone)-b-D-α-tocopheryl polyethylene glycol 2000 succinate nanoparticles for lung cancer therapy. Int. J. Nanomed..

[57] Zhou Z. (2020). Co-drug delivery of regorafenib and cisplatin with amphiphilic copolymer nanoparticles: Enhanced in vivo antitumor cancer therapy in nursing care. Drug Deliv..

[58] Wu L., Leng D., Cun D., Foged C., Yang M. (2017). Advances in combination therapy of lung cancer: Rationales, delivery technologies and dosage regimens. J. Control. Release.

[59] Shendge R.S., Sonawane P.S., Khade S.B. (2026). Innovations in nanoparticle-based drug delivery for lung cancer: Recent developments and future horizons. Nanomed. J..

