# Supplementary material for: Nanocarrier-Based Drug Delivery Systems for Lung Cancer: A Systematic Review and Meta-Analysis of Preclinical Studies
Source: Adv Respir Med. 2026 Jul 24;94(4):50. doi: 10.3390/arm94040050 (PMC13509827; doi:10.3390/arm94040050)
Supplement: Supplementary file 1 [file arm-94-00050-s001.zip › arm-4416185-supplementary.pdf]

SUPPLEMENTARY MATERIAL

Nanocarrier-Based Drug Delivery Systems for Lung Cancer: A Systematic Review and Meta-Analysis of Preclinical Studies

Running title: Drug delivery systems for lung cancer: a meta-analysis

CONTENTS

Table S1. PECOS model

Table S2. The search and screening strategy

Figure S1. Flow chart of studies included in meta-analysis

Table S3. Main characteristics of included studies.

Figure S2. Efficacy of chemotherapeutics in DDS in inhibiting tumor growth compared to the free drug as a control group

Figure S3. Efficacy of chemotherapeutics delivered via targeted and non-targeted drug delivery systems compared to free chemotherapeutics on tumor growth inhibition

Figure S4. Efficacy of chemotherapeutics delivered via targeted versus non-targeted drug delivery systems on tumor growth inhibition

Figure S5. Efficacy of chemotherapeutics delivered via drug delivery systems compared to free chemotherapeutics on tumor growth inhibition: Subgroup analysis of Cisplatin, Paclitaxel, Docetaxel, and Doxorubicin.

Figure S6. Efficacy of combination of two chemotherapeutics delivered via drug delivery systems compared to free chemotherapeutics on tumor growth inhibition: Subgroup analysis of combination of two cytostatic drugs Cisplatin+ Paclitaxel, and Cisplatin + Docetaxel.

Figure S7. Efficacy of chemotherapeutics in DDS compared to free chemotherapeutics on tumor growth inhibition in subgroup analysis: Intravenous, Intraperitoneal, and Intratumoral.

Figure S8. Efficacy of chemotherapeutics in various DDS types compared to free chemotherapeutics on tumor growth inhibition in subgroup analysis: Nanoparticles, Liposomes, and Micelles

Table S4. Assessment of risk of bias in the included studies using SYRCLE's RoB tool for animal studies

Table S1. PECOS model

PECOS model	
**Participants**	Experimental lung cancer models in mice (any strain and sex). Tumor models were established using human or murine lung cancer cell lines including A549, NCI-H82, M109, H446, H460, H2009, H358, L132, and Lewis lung carcinoma (LLC)
**Exposure:**	Administration of chemotherapeutic agents encapsulated in drug delivery systems (DDS), including liposomes, polymeric nanoparticles, micelles, or other nanocarrier-based formulations evaluated for lung cancer treatment in animal models
**Comparator/Control**	Animals receiving the corresponding free (non-encapsulated) chemotherapeutic agent or untreated control groups
**Outcomes**	Primary outcome: tumor growth inhibition measured as tumor volume. Secondary outcomes included survival rate and treatment response indicators reported in the included studies.
**Study design**	Controlled in vivo experimental studies in animal models evaluating the therapeutic efficacy of DDS-encapsulated chemotherapeutic agents for lung cancer treatment. Reviews, meta-analyses, editorials, and in vitro studies were excluded.

Table S2. Literature search strategy

**Electronic databases**	PubMed/MEDLINE, EMBASE, Scopus, Google Scholar, the Cochrane Central Register of Controlled Trials (CENTRAL), and ClinicalTrials.gov were searched from
**Search strategy (PubMed/EMBASE example**	("drug delivery system*" OR nanoparticle* OR liposome* OR micelle* OR polymeric nanoparticle* OR PLGA OR PLA OR PCL OR PEO OR targeted DDS OR non-targeted DDS) AND (cisplatin OR carboplatin OR paclitaxel OR docetaxel OR gemcitabine OR etoposide OR vinorelbine) AND ("lung cancer" OR "non-small cell lung cancer" OR NSCLC) AND ("tumor growth inhibition" OR survival OR antitumor activity)
Animal model terms	mouse OR mice OR murine OR xenograft OR animal model
**Outcome terms**	efficacy OR safety OR tumor growth inhibition OR survival rate
**Additional search**	American Association for Cancer Research (AACR), the European Society for Medical Oncology (ESMO), the American Cancer Society (ACS), and Cancer Research UK.

https://pubmed.ncbi.nlm.nih.gov/?term=((((((((((((((((((((Nanoparticles)%20OR%20(Drug%20delivery%20systems))%20OR%20(Liposomes))%20OR%20(Polymer))%20OR%20(Micelles))%20OR%20(PCL))%20OR%20(PLA))%20OR%20(PLGA))%20OR%20(PEO))%20OR%20(Targeted))%20OR%20(Non-targeted))%20AND%20(cisplatin))%20OR%20(carboplatin))%20OR%20(docetaxel))%20OR%20(etoposide))%20OR%20(paclitaxel))%20OR%20(gemcitabine))%20OR%20(vinorelbine))%20AND%20(Survival))%20OR%20(growth%20inhibition))%20AND%20(lung%20cancer)&sort=relevance

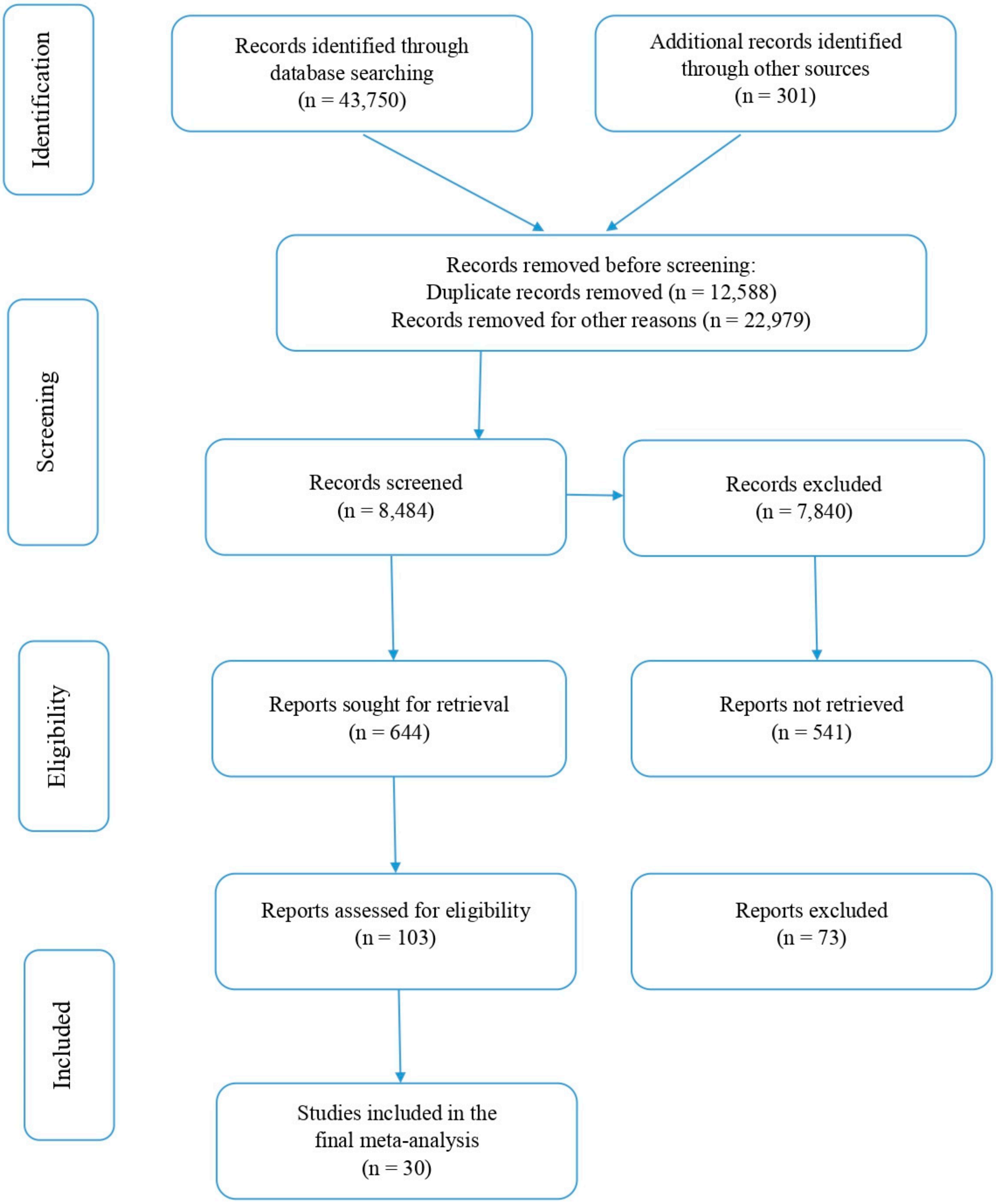

Figure S1. Flow chart of studies included in meta-analysis

Table S3. Main characteristics of included studies.

Nr	Auth Year	Type DDS	Cytostatic Drug	Targeting (active/ passive)	Species /strain / genotype	No per group	Cell type (no. of cells)	Inoculation area	Administration route	Outcome measures
1	Cao, 2016 (28)	LNp	cisplatin	Passive	Female BALB/c nude mice	5 groups (n =4 per group)	A549R 5×106	into the right flank	IV	Tumor volume in mm3<br>PBS 750: CISPlatine 500; CLAN0% 480: CLAN 1% 300; CLAN 3% 200
2	Guo, 2019 (29)	LNP	Paclitaxel	Active	Female BALB/c nude mice	10 group(6 mice)	A549 $5x10^6$	s.c	IV	Tumor volume in mm3<br>1/ CET-PTX/DMN-NLCs 230<br>2 PTX/DMN-NLCs ~300<br>3 PTX-NLCs~400<br>4 DMN-NLCs ~450<br>5 CET-NLCs~350<br>6 Free PTX/DMN ~600<br>7 Free PTX ~700<br>8 Free DMN ~800<br>9 Blank NLCs ~950<br>10 0.9% Saline ~1050
3	He, 2015 (30)	NP	Cisplatin and Paclitaxel	Active	Male, Balb-c/nude mice,	ND	A549 and M109 cells ($1×10^6$ cells, 0.1 mL in PBS)	s.c transplanted into the right flank of male Balb-c/nude mice,	IV	Tumor volume in mm3 in A549:PBS1750; free-PTX 800 , free-CDDP 620, CDDP-FA-NPs 450,<br>PTX-FA-NPs 550, Co-FA-NPs (CDDP: PTX = 1:2) 150; Co-NPs(CDDP: PTX=1:2) 200 and free-CDDP<br>+ free-PTX 350 Tumor volume in mm3 for M109 :PBS1700; free-PTX 850 , free-CDDP 600, CDDP-FA-NPs 250,<br>PTX-FA-NPs 200, Co-FA-NPs (CDDP: PTX = 1:2) 58; Co-NPs(CDDP: PTX=1:2) 100 and free-CDDP<br>+ free-PTX 300
4	Iyer,2020 (31)	NP	Cisplatin (CDDP)	Pasisive	CGPUs, athymic nude mice	6 mice per group (3 males and 3 females) 4 groups	A549 (2 million cells)	sc on hind-limb	IV	Tumor volume in mm3<br>Saline 2315; GPU 2487; Cis 1372; CGPU 343
5	Jin,2016 (32)	NP	Docetaxel	Passive	Female BALB/c mice	4 group mice (n = 10)	A549;$3x 10^6$	alar or in the lung.	IV	Tumor volume in mm3<br>Control 2100;<br>DANPs 750;<br>DTX 1350;<br>PEG DANPs 150
6	Jin,2020 (33)	NP	cisplatin (CDDP) and Docetaxel	Passive	Female BABL/c mice	7 groups (8 mice per group)	A549/CIS cells (100 µL, contained $1 × 10^6$ cells)	subcutaneous injected to the right flank of mice	IV	Tumor volume in mm3<br>CIS-DOXp NC 250; CIS-DOX NC (CIS 5 mg, DOX 15 mg) 400, (3) CIS NC (CIS 10 mg)500, (4) DOX NC (DOX 30 mg)550, (5) free CIS-DOX (CIS 5 mg, DOX 15 mg)820, (6) NC1300, and (7) normal saline (NS)1250
7	Jung, 2012 (34)	NP	Paclitaxel and Docetaxel	Passive	male BALB/c nude mice (	(n=5/ group).	A549 cells ($4 10^5$ cells)	implanted subcutaneously into the right hind legs of	iv	Tumor volume in mm3<br>Controll 480; Docetaxel 250; PNPDTX 190 mm3; IR 350; PNPDTX + IRand PNP DTX+IR 100 mm3,

8	Li, 2011 (35)	NP	Paclitaxel	Active	Female NOD/SCID mice	5 mice per groupe	1 million H2009 (Human lung cancer cell lines) cells	in the right flank	IV	Tumor volume in mm3<br>controle (salin) 1400;<br>free drug PTX 1150;<br>H2009.1 -PTX 1250
9	Li, 2020 (36)	NP	Docetaxel	Active	C57BL/6	6 group (6 mice)	LLC 1 $\times 10^7$ mL−1	s.c.	IV	Tumor volume in mm3<br>controle (salin) 1750;<br>free drug DTX 1150;<br>DMS 800; FREE combo 500<br>PDMS 300<br>PDM+US 250
10	Liang,2015 (37)	NP	docetaxel	active	male nude mice	4 groupe (8 mice)	H446 1×10^6 cells/100 µL	Subcutaneous tumors on the right flank of male nude mice	IP	Tumor volume in mm3<br>Controle (saline) 1050mm3;<br>DTX 690;<br>DN 640;<br>AP-DN 520
11	Long, 2014 (38)	NP	Doxorubicin	Active	BALB/c nu/nu mice female	4 group<br><br>(4 mice)	A549 1,5X10^6		IN	Tumor volume in mm3<br>Control 800;<br>Doxorubicin 500;<br>GNP 400;<br>EGNP 150.
12	Nguyen,2001 (39)	LPN	Paclitaxel	passive	nude mice	8 (for each gropup)	10^7 cells suspended H358 human tumor in 100 mL of phosphate-buffered saline	in hind flanks of nude mice	IP	Group/tumor volume at 8 weeks (mm3) :<br>Control / 2,500 mm3;<br>17-AAG (10 mg/kg) / 2,250 mm3;<br>17-AAG (25 mg/kg) /1,250 mm3<br>Paclitaxel (1 mg/kg)/ 1,200 mm3<br>17-AAG (10) + paclitaxel 700 mm3<br>17-AAG (25) + paclitaxel/350 mm3
13	Shi, 2023 (40)	NP	Paclitaxel	Acitve	Male nude mice	5 groups /(10 mice (total 50)	1×10^6 A549 cells	right leg of nude mice.	IV	Tumor growth volume in mm^3:<br>NS ~900;<br>Bif@PDA-NPs: ~950;<br>Free PTX : ~500;<br>PDA-PTX-NPs: ~350;<br>Bif@PDA-PTX-NPs: ~150.
14	Shuhang Wang ,2021 (41)	Micelle	cisplatin (CDDP)	Passive	Male C57BL/6 mice	5 gropus	LLC 1x10^7	Right flank	IV	saline 1200mm3;<br>liposome 600mm3;<br>NVB liposome 550mm3;<br>CoNP(CDDP+NVB) 250mm3;<br>andM(PTX0.3/Pt)150mm3.
15	Song,2014 (42)	Micelle	paclitaxel (PTX) and cisplatin	Passive	Balb/C nude mice male	5 groups (n =6 per group)	1.5 10^6 A549 cells	sc into right flank of each mous	IV	Tumor volume in mm3<br>saline 1000mm3; PTX400mm3;<br>PTX+CDDP200mm3;<br>M(PTX)300mm3;<br>andM(PTX0.3/Pt)150mm3.
16	Tang, 2010 (43)	NP	Etoposide	passive	female Hsd:Athymic NudeFoxn1 nu mice	ND	1×10^6 NCI-H82 cells suspended in 50% Matrigel	s.c. in the right flank	IT	on 10 day:<br>Blank PEG-PSA particles 850 mm^3<br>IP-Free etoposide 800 mm^3<br>IT Free etoposide 750 mm^3<br>IT-Etoposide in PEG-PSA NP 80 mm^3
17	Wang,2018 (44)	LNp	Paclitaxel and cisplatine	Passive	Balb/c-nude mice	8 mice per group	A549 cells (10^7)	scsubcutaneously injected into the right flank of mice	IV	Tumor volume in mm3<br>RGD-ss-PTX/CDDP LPNs; 300;<br>RGD-ss-PTX LPNs 600;RGD CDDP LPNs 700; PTX/CDDP LPNs 800; RGD LPNs1600;<br>PTX/CDDP LPNP800; free PTX1200; Free CDDP 1350;<br>Saline 1900
18	Wang, 2021 (45)	Np exosoms	docetaxel	Passive	Balb/c nude mice	5groups (6 mice)	A549 (1 10^7	sc in the left legs of the mouse	IV	Tumor volume in mm3<br>Control 1150;<br>EXO 950;<br>DTX 600;<br>EXo-DTX 300

19	Wang, 2025 (46)	LNP	Cisplatin	passive	BALB/c nude mice, male	5 groups (n =5 per group)	A549-DDP (5 ×10^6	s.c.into the right flanks	PT	Tumor volume in mm3<br>controle 680mm3;<br>DDP 650mm3;<br>21CP_8K_4 + PLGA-DDP@LB 600mm3; PLGA-DDP@LB-CP_8E_4 620mm3;<br>CP_8K_4 + PLGA-DDP@LB-CP_8E_4 130 mm3
20	Wu, 2020(47)	LpNP	docetaxel prodrug (DTXp) and cisplatin (DDP)	Active	Female BALB/c mice (Charles River, Arbresle, France)	7 groups (6 mice per group).	A549 cells (7×10^6 , 0.2 mL per mice)	s.c into the flanks of nude mice	IV	Tumor volume: Control ~1500 mm^3;<br>Free DTX/DDP: ~1100 mm^3;<br>APT-DDP-LPHNs: ~850 mm^3;<br>APT-DTX-LPHNs;~900 mm^3;<br>APT-DTXp-LPHNs: ~550 mm^3;<br>DTXp/DDP-LPHNs: ~500 mm^3;<br>APT-DTXp/DDP-LPHNs: ~250 mm^3
21	Xiong, 2016 (48)	NP	cisplatin (CDDP)	Active	female nude mice (Harlan Sprague Dawley)	5 groups (n = 4–6)	5 × 10^6 H460 cells in 100 µL of (PBS)	s.c.into the right flank	IV	Tumor volume in mm3<br>untreated control (PBS) 1500,<br>free CDDP 200,<br>polymet NPs 300,<br>polymetCDDP NPs 130 and<br>PEI-CDDP NPs350
22	Yang, 2012 (49)	NP	Paclitaxel	- passive	female athymic nu/nu nude mice	3 mice per group (3 groups)	NCI-H460 cells s 4 × 10^6 cells	sc on right-limb armpit of mice	IV	Tumor volume in mm3<br>vehicle pbs 2700;<br>PTX 2000;<br>PGG PTX 1200
23	Yang, 2023 (50)	NP	Cisplatin	Active	BALB/CAnN.Cg-Foxn1nu/CrlNarl nude mice	5 groups (n=5 in each group): total25	A549 (5 5×10^6	sc.front flank (distal tumor) and the hind flank close to the leg (primary tumor)	IV	Tumor volume in mm^3 at 28 days:<br>saline 700mg,<br>free CDDP 500mg<br>HP@GNSs +NIR group 350<br>HCP@GNSs 250<br>HCP@GNSs +NIR group 50
24	Yuan, 2015 (51)	Micelle	Paclitaxel	passive	Female nude mice	(n = 5)	5×10^6 luc-A549	inoculated into the upper lobe of the right lung	IV	Tumor volume in mm3<br>Saline 780mm3;<br>Taxol 550;<br>PTX/SPCS 300;<br>PTX/ cRGDyK- SPCS 150
25	Yuan,2016 (52)	NP	Paclitaxel	Passive	Male Balb/c nude mice,	4 groups (n=10)	5×10^6 A549 cells	subcutaneously into the right armpit of male	IV	Tumor volume in mm3<br>Saline 300; Taxol,270 PTX-loaded PLGA NPs252.6, and PTX-loaded<br>PLGA-Tween 80 NPs 238.5
26	Yu-Yang Bi, 2024 (53)	NP	cisplatine	Active	Female and male BALB/c nude mice.	7 groups (n=5) total 35mice	p53 mutant human NSCLC cell lines NCI-H1975 (1.0×10^7 /mL)	s.c	IV	Tumor volume in mm3:<br>PBS~2300;<br>Flu~2100;<br>Cis~2000;<br>Flu+Cis~1900;<br>F+C NPs~1300<br>F NPs~250;<br>FP NPs~107.
27	Zelai He, 2015 (54)	NP	CDDP and PTX	acitve	male Balb-c/nude mice.	8groupe (n = 6)	A549 and M109 cells (1 × 10^6 cells in 0.1 mL of PBS)	subcutaneously inoculated into the right flank	IV	Tumor volume in mm3 for A549 :PBS1750; free-PTX 800 , free-CDDP 620, CDDP-FA-NPs 450, PTX-FA-NPs 550, Co-FA-NPs (CDDP: PTX = 1:2) 150; Co-NPs(CDDP: PTX=1:2) 200 and free-CDDP<br>+ free-PTX 350<br><br>Tumor volume in mm3 for M109 :PBS1700; free-PTX 850 , free-CDDP 600, CDDP-FA-NPs 250, PTX-FA-NPs 200, Co-FA-NPs (CDDP: PTX = 1:2) 58; Co-NPs(CDDP: PTX=1:2) 100 and free-CDDP<br>+ free-PTX 300

28	Zhang,2018 (55)	Micelle	Paclitaxel	passive	BALB/c nu/nu mice female	5 group (6 mice)	A549/PTX cells (1 × 10^7/0.1 mL)	s.c.	IV	Tumor volume in mm3<br>Saline 1000 mm3 ;<br>IM (15 mg kg−1 ITA) 600 mm3,<br>PM/IM (mix micelle ITA or PTX 15 mg kg−1) 550 mm3, and<br>PIM (ITA andPTX 15 mg kg−1 micelle) 200mm3
29	Zhou, 2013 (56)	NP	Paclitaxel	passive	nude mice	3 groups (each group, n=5)	A-549 cells (0.1 mL)of 2 × 10^7 cells/mouse	sc on right-limb armpit of mice	IT	Tumor volume in mm3<br>controle (salin) 550;<br>free drug PTX 300; PTX(PGA-co-PCL)-b-TPGS2k NPs 150
30	Zhou, 2020 (57)	NP	Regorafenib (REGO) and Cisplatin (PT)	Active	BALB/c nude mice	5 groups (n= 10 mice per group)	A549 (2.5 x10^7 cells/mL)	s.c.into the right flank	IV	Tumor volume in mm3<br>Saline (Control): ~2500 mm³<br>Free PT (2.5 mg/kg): ~1400 mm³l<br>Free REGO (2.5 mg/kg): ~1000 mm³<br>PT NPs (5 mg/kg) : ~800 mm³<br>REGO NPs (5 mg/kg): ~600 mm³<br>PT-REGO NPs (10 mg/kg): ~300 mm³

Abbreviation

Cisplatin CDDP; Paclitaxel PTX; Docetaxel DTX; Doxorubicin DOX; Etoposide- Etop**;** Non small cell lung cancer (NSCLC); Nanoparticle –NP; lipid nanoparticle – LNP; intratumoral – IT; Intravenous – IV; intraperitoneal –IP; inhalation –IN;PT- peritumoral; subcutaneously- s.c; phosphate-buffered saline (PBS); polyglutamic acid (PGA); DOTAP (2, 3-Dioleoyloxy-propyl)trimethylammonium/Cholesterol/DSPE-PEG-anisamide aminoethyl; docetaxel-loaded PEG-albumin nanoparticles (PEG-DANPs); PLGA, poly(d,l-lactide-co-glycolide); 4methylumbelliferone (4Mu) Hyaluronan (HA); CoNP-lip, vinorelbine and cisplatin co-delivery liposome;Plga, poly(d,l-lactide-co-glycolide); Dcc, N,N′-dicyclohexylcarbodiimide; Nhs, N-hydroxysuccinimide; 17-allylamino geldanamycin (17-AAG); Doxorubicin DOr, EGF- epidermal growth factor, EGFR modified epidermal growth factor gelatin nanoparticles, CLANs – Cationic Lipid–Assisted Nanoparticles; CLAN-0% – CLANs with 0% cRGD modification; CLAN-1% – CLANs with 1% cRGD modification; CLAN-3% – CLANs with 3% cRGD modification; PBS – Phosphate Buffered Saline; A549R – Cisplatin-resistant A549 lung cancer cells; PTX – Paclitaxel; GNP – Gelatin Nanoparticle; PTX-GNP – Paclitaxel-loaded Gelatin Nanoparticles; PEG-PTX-GNP – Polyethylene glycol-modified Paclitaxel-loaded: Gelatin Nanoparticles; NPs – Nanoparticles; BALB/c – Albino, immunocompetent mouse strain; L132 – Human lung epithelial cell line; DMN – Demethoxycurcumin; NLCs – Nanostructured Lipid Carriers; CET – Cetuximab; CET-NLCs – Cetuximab-modified Nanostructured Lipid Carriers; PTX/DMN-NLCs – Nanostructured lipid carriers co-loaded with PTX and DMN; CET-PTX/DMN-NLCs – Dual-drug-loaded NLCs further modified with CET; Blank NLCs – Drug-free nanostructured lipid carriers; TV – Tumor Volume; FA-NPs – Folic acid-modified nanoparticles;Co-FA-NPs – Dual drug-loaded FA-modified nanoparticles (CDDP + PTX); Co-NPs – Dual drug-loaded nanoparticles without FA; M109 – Murine lung carcinoma cells; DLC – Drug Loading Content.; GPUs – Gelatin-based Nanoparticles (non-conjugated); CGPUs – Cisplatin-loaded Gelatin-based Nanoparticles (conjugated); TGR – Tumor Growth Rate; PFA – Paraformaldehyde; PBS – Phosphate Buffered Saline (saline control); DANPs – Docetaxel-loaded Albumin Nanoparticles; PEG-DANPs – PEGylated Docetaxel-loaded Albumin Nanoparticles; Aisu® – Commercial docetaxel formulation (positive control);; NC – Nanocarrier; CIS NC – Nanocarrier loaded with cisplatin;

DOX NC – Nanocarrier loaded with doxorubicin; CIS-DOX NC – Nanocarrier co-loaded with CIS and DOX; CIS-DOXp NC – Optimized co-loaded nanocarrier (formulation with superior efficacy); NS – Normal saline (negative control); A549/CIS – Cisplatin-resistant human lung adenocarcinoma cells; PNP-PTX / PNP-DTX – Polymeric nanoparticle formulations of paclitaxel and docetaxel; IR – Ionizing radiation (5 Gy, 6-MV beam);T/C (%) – Tumor/control ratio: % of tumor volume change in treated; H2009 – Human non-small cell lung cancer cell line**;** H2009.1–PTX – Tetrameric H2009.1 peptide–paclitaxel conjugate; NOD/SCID mice – Non-obese diabetic/severe combined immunodeficient mice.; LLC; Lewis Lung Carcinoma; DMs; Docetaxel-loaded Microbubbles; PDMs; PD-L1 antibody and Docetaxel-loaded Microbubbles; US; Ultrasound irradiation; CT-Computed Tomography; PD-L1 mAb- Programmed Death-Ligand 1 monoclonal antibody; GNP -gelatin-based nanoparticles; EGNP- EGF-conjugated gelatin-based nanoparticles; EGF- epidermal growth factor; 17-AAG **-** 17-allylamino-17-demethoxygeldanamycin**;** H358 human non-small cell lung carcinoma (NSCLC)**;** VEGF vascular endothelial growth factor**;** ErbB2 (HER2) **-** human epidermal growth factor receptor 2; Caspase-3 **-** executioner protease **;** PDA – Polydopamine; PDA-PTX-NPs – Polydopamine nanoparticles loaded with paclitaxel; Bif – *Bifidobacterium* (a probiotic bacterium used for tumor targeting); Bif@PDA-NPs – *Bifidobacterium*-coated polydopamine nanoparticles;Bif@PDA-PTX-NPs – *Bifidobacterium*- coated polydopamine nanoparticles loaded with paclitaxel (biohybrid system); EXO-DTX: Exosome-loaded docetaxel formulation; EXO: Exosome (natural nanoscale extracellular vesicles);: Normal saline (control group)LLC**:** Lewis Lung Carcinoma; NVB: Vinorelbine; NP-sol**:** Nanoparticle solution (non-encapsulated drug); CDDP-lip: Liposome-encapsulated Cisplatin; NVB-lip**:** Liposome-encapsulated Vinorelbine; CoNP-lip**:** Co-delivered Cisplatin and Vinorelbine in liposomes**.;** SPF: Specific Pathogen-Free**;** M(PTX)**:** Micelles loaded with paclitaxel; M(PTX0.3/Pt)**:** Micelles co-loaded with paclitaxel and cisplatin at a ratio of 0.3; TSR%**:** Tumor Suppression Rate (relative to control); TGR%**:** Tumor Growth Rate; PEG-PSA: Poly(ethylene glycol)-co-poly(sebacic acid);; Etop/PEG-PSA: Etoposide-loaded PEG-PSA polymer particles; NCI-H82: Human small cell lung cancer cell line; LPNs: Lipid–polymer hybrid nanoparticles; RGD: Arginine–glycine–aspartic acid peptide (targeting moiety); RGD-ss-PTX/CDDP LPNs: Redox-sensitive, dual drug-loaded lipid–polymer hybrid nanoparticles functionalized with RGD peptide;RGD-ss-PTX LPNs: PTX-loaded LPNs with disulfide linkage and RGD; RGD-CDDP LPNs: CDDP-loaded LPNs with RGD; PTX/CDDP: Combination of free paclitaxel and cisplatin; TV: Tumor volume; SPF: Specific pathogen-free;IP: Intraperitoneal; IV: Intravenous.; EXO-DTX**:** Exosome-loaded docetaxel formulation**;** EXO**:** Exosome (natural nanoscale extracellular vesicles)**;** MF-DDS**:** Magnetically targeted functional drug delivery system; A549-DDP: Cisplatin-resistant human lung adenocarcinoma cell line; PLGA**:** Poly(lactic-co-glycolic acid), a biodegradable polymer used for nanoparticle formulation; CP8K4 / CP8E4**:** Peptides used for enhancing tumor targeting and cell penetration; APT**:** Aptamer (tumor-targeting ligand); DTXp: Docetaxel prodrug; LPHNs**:** Lipid–polymer hybrid nanoparticles; APT-DTXp/DDP-LPHNs: Lipid–polymer hybrid nanoparticles co-loaded with docetaxel prodrug and cisplatin, decorated with aptamer; APT-DTXp-LPHNs: LPHNs containing docetaxel prodrug and aptamer; APT-DDP-LPHNs: LPHNs containing cisplatin and aptamer; DTXp/DDP**-**LPHNs: Non-decorated (no aptamer) co-loaded nanoparticles;); PEI**:** Polyethylenimine (a polymer used for gene/drug delivery); Polymet**:** Polymeric materials (unspecified in this section, likely ;cationic polymers for drug delivery); Polymet-CDDP NPs**:** Nanoparticles formed by complexing polymet with cisplatin**;** PEI-CDDP NPs: Nanoparticles combining PEI and cisplatin; H460: Human NSCLC cell line used for xenograft model; NCI-H460: Human non-small cell lung cancer cell line; PGG**:** Poly(L-glutamic acid-co-L-glutamate) (polymer carrier for paclitaxel); PGG–PTX: Conjugate of

poly(L-glutamic acid) with paclitaxel (PTX); HCP@GNSs-CDDP-loaded hybrid chitosan/gold nanoshells; HP@GNSs- Hybrid polymer/gold nanoshells (without drug); cRGDyK- Cyclic peptide targeting integrin receptors; PTX/cRGDyK-SPCS micelles- Micelles loaded with paclitaxel and modified with cRGDyK for targeted delivery; PIM- Paclitaxel and ITA Co-encapsulated Micelles; PM-Paclitaxel Micelles; IM-ITA itraconazole Micelles; PM/IM- Physical mixture of PM and IM; A549/PTX-Paclitaxel-Resistant A549 Cell Line; ITA- itraconazole; P-gp-P-glycoprotein. DN Docetaxel-loaded nanoparticles; AP-DN Asialoglycoprotein receptor-targeted DN; PGA-co-PCLP-oly(glycolide-co-ε-caprolactone); TPGS2k- D-α-Tocopheryl polyethylene glycol 2000 succinate; PGA-co-PCL)-b-TPGS2k Block copolymer of PGA-co-PCL with TPGS2k; REGO- Regorafenib; PT NPs- Paclitaxel-loaded nanoparticles; REGO NPs-Regorafenib-loaded nanoparticles; PT-REGO NPs-Dual-loaded nanoparticles with paclitaxel and regorafenib; PLGA- Poly(lactic-co-glycolic acid) – a biodegradable polymer for drug delivery; Tween 80-Polyoxyethylene sorbitan monooleate – a surfactant to modify nanoparticle surfaces; Taxol- Commercial formulation of paclitaxel; FP NPs- Fluvastatin and Cisplatin; F NPs-Fluvastatin-loaded Nanoparticles; H1975- Human NSCLC cell line with EGFR and p53 mutation ; FA-NPs-Folic Acid-modified Nanoparticles; Co-NPs- Nanoparticles co-loaded with CDDP and PTX; Co-FA-NPs-Folic Acid-modified co-loaded Nanoparticles;

Study or Subgroup	DDS+ Drug Mean	DDS+ Drug SD	DDS+ Drug Total	Free Drug Mean	Free Drug SD	Free Drug Total	Weight	Mean Difference IV, Random, 95% CI
Cao, 2016	326.7	157.12	12	500	248	4	2.3%	-173.30 [-432.08, 85.48]
Guo, 2019	265	126.5	12	600	297	6	2.4%	-335.00 [-583.19, -86.81]
Guo*,2019	400	197	6	700	347	6	2.0%	-300.00 [-619.28, 19.28]
He, 2015	450	222	6	620	307	6	2.0%	-170.00 [-473.14, 133.14]
He*,2015	550	272	6	800	397	6	1.6%	-250.00 [-635.07, 135.07]
He comb,2015	175	81.5	12	350	172	6	3.1%	-175.00 [-320.15, -29.85]
He combM109,2015	79	33.5	12	300	147	6	3.3%	-221.00 [-340.14, -101.86]
He M109,2015	250	122	6	600	297	6	2.3%	-350.00 [-606.91, -93.09]
He M109*,2015	200	97	6	850	422	6	1.8%	-650.00 [-996.47, -303.53]
Iyer, 2020	343	168.5	6	1,372	683	6	1.0%	-1029.00 [-1591.89, -466.11]
Jin, 2016	150	70	10	1,350	670	10	1.5%	-1200.00 [-1617.52, -782.48]
Jin, 2020	425	196.5	32	820	406	8	2.1%	-395.00 [-684.46, -105.54]
Jung, 2012	190	92.5	5	250	122.5	5	3.2%	-60.00 [-194.55, 74.55]
Jung*,2012	200	97.5	5	220	107.5	5	3.2%	-20.00 [-147.21, 107.21]
Li,2011	950	472.5	5	1,150	572.5	5	0.8%	-200.00 [-850.64, 450.64]
Li,2020	275	131.5	12	1,150	572	6	1.3%	-875.00 [-1338.69, -411.31]
Ling, 2015	580	282	16	690	341	8	2.2%	-110.00 [-383.73, 163.73]
Long,2014	275.8	133.5	8	500	248	4	2.3%	-224.20 [-484.25, 35.85]
Nguyen,2001	550	267	16	1,200	596	8	1.4%	-650.00 [-1083.23, -216.77]
Shi, 2023	250	115	20	500	245	10	3.0%	-250.00 [-410.00, -90.00]
Shuhang Wang, 2021	250	122	6	575	281.5	12	2.8%	-325.00 [-511.81, -138.19]
Song, 2014	150	72	6	200	97	6	3.4%	-50.00 [-146.66, 46.66]
Tang, 2010	80	37	6	750	372.5	5	1.9%	-670.00 [-997.84, -342.16]
Wang, 2018	300	146	8	950	471	8	1.8%	-650.00 [-991.70, -308.30]
Wang,2021	300	147	6	600	297	6	2.3%	-300.00 [-565.16, -34.84]
Wang,2025	410	197.5	15	550	272.5	5	2.3%	-140.00 [-398.92, 118.92]
Wang*,2018	700	346	8	1,350	671	8	1.1%	-650.00 [-1173.15, -126.85]
Wang**,2018	600	296	8	1,200	596	8	1.3%	-600.00 [-1061.13, -138.87]
Wu, 2020	375	181.5	12	1,100	547	6	1.3%	-725.00 [-1174.57, -275.43]
Wu*,2020	767	380.5	18	1,100	547	6	1.3%	-333.00 [-804.66, 138.66]
Xionga, 2016	215.12	101.5	12	200	97	6	3.4%	15.12 [-81.43, 111.67]
Yang, 2012	1,200	598.5	3	2,000	998.5	3	0.2%	-800.00 [-2117.32, 517.32]
Yang, 2023	350	172.5	5	500	247.5	5	2.3%	-150.00 [-414.43, 114.43]
Yuan, 2015	225	107.5	10	550	272.5	5	2.4%	-325.00 [-572.97, -77.03]
Yuan, 2016	248.25	114.125	20	270	130	10	3.4%	-21.75 [-116.59, 73.09]
Yu-Yang Bi, 2024	552.3	268.65	15	1,900	947.5	5	0.5%	-1347.70 [-2189.26, -506.14]
Zelai He, 2015	300	147	6	550	272	6	2.4%	-250.00 [-497.39, -2.61]
Zelai He*,2015	400	197	6	750	372	6	1.9%	-350.00 [-686.82, -13.18]
Zelai He comb,2015	135	61.5	12	250	122	6	3.4%	-115.00 [-218.63, -11.37]
Zelai He combM109,2015	85	36.5	12	350	172	6	3.2%	-265.00 [-404.17, -125.83]
Zelai He M109, 2015	250	122	6	600	297	6	2.3%	-350.00 [-606.91, -93.09]
Zelai He M109*,2015	300	147	6	900	447	6	1.6%	-600.00 [-976.51, -223.49]
Zhang,2018	375	181.2	12	700	347	6	2.1%	-325.00 [-620.98, -29.02]
Zhou,2013	150	72.5	5	300	147.5	5	3.1%	-150.00 [-294.06, -5.94]
Zhou, 2020	800	395	10	1,400	695	10	1.2%	-600.00 [-1095.47, -104.53]
Zhou*,2020	600	295	10	1,000	495	10	1.7%	-400.00 [-757.15, -42.85]
ZhouComb,2020	567	268.5	30	1,200	595	20	2.2%	-633.00 [-910.90, -355.10]
**Total (95% CI)**			**486**			**318**	**100.0%**	**-310.67 [-375.51, -245.83]**

Heterogeneity: Tau² = 29514.61; Chi² = 169.02, df = 46 (P < 0.00001); I² = 73%
Test for overall effect: Z = 9.39 (P < 0.00001)

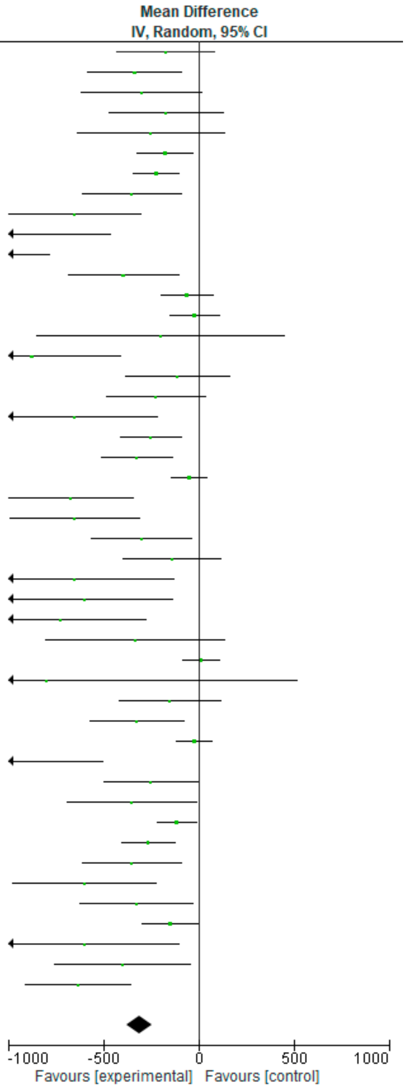

Figure S2. Efficacy of chemotherapeutics in DDS in inhibiting tumor growth compared to the free drug as a control group

Study or Subgroup	Targeted/Non Targeted DDS + Drug Mean	SD	Total	Free Drug Mean	SD	Total	Weight	Mean Difference IV, Random, 95% CI
**2.2.1 Targeted DDS + Drug Vs. Free Drug**								
Cao, 2016	326	157.35	12	500	248	4	1.9%	-174.00 [-432.83, 84.83]
Guo, 2019	230	112	6	700	347	6	1.7%	-470.00 [-761.76, -178.24]
He, 2015	450	222	6	620	307	6	1.7%	-170.00 [-473.14, 133.14]
He*,2015	550	272	6	800	397	6	1.3%	-250.00 [-635.07, 135.07]
He comb,2015	150	72	6	350	172	6	2.7%	-200.00 [-349.20, -50.80]
He combM109,2015	58	26	6	300	147	6	2.8%	-242.00 [-361.45, -122.55]
He M109,2015	250	122	6	600	297	6	2.0%	-350.00 [-606.91, -93.09]
He M109*,2015	200	97	6	850	422	6	1.5%	-650.00 [-996.47, -303.53]
Li,2011	950	472.5	5	1,150	572.5	5	0.6%	-200.00 [-850.64, 450.64]
Li,2020	300	147	6	1,150	572	6	1.0%	-850.00 [-1322.56, -377.44]
Ling, 2015	520	256	8	690	341	8	1.7%	-170.00 [-465.47, 125.47]
Long,2014	150	73	4	500	273	4	1.8%	-350.00 [-626.93, -73.07]
Shi, 2023	150	70	10	500	245	10	2.6%	-350.00 [-507.93, -192.07]
Wang, 2018	800	396	8	950	471	8	1.1%	-150.00 [-576.41, 276.41]
Wang,2025	130	62.5	5	550	272.5	5	2.0%	-420.00 [-665.05, -174.95]
Wang*,2018	700	346	8	1,350	671	8	0.9%	-650.00 [-1173.15, -126.85]
Wang**,2018	600	296	8	1,200	596	8	1.0%	-600.00 [-1061.13, -138.87]
Wu, 2020	250	122	6	1,100	547	6	1.1%	-850.00 [-1298.44, -401.56]
Yuan, 2015	150	72.5	5	550	272.5	5	2.0%	-400.00 [-647.16, -152.84]
Yuan, 2016	238.5	114.25	10	270	130	10	2.9%	-31.50 [-138.77, 75.77]
Yu-Yang Bi, 2024	107	51	5	1,900	947.5	5	0.4%	-1793.00 [-2624.71, -961.29]
Zelai He comb,2015	120	57	6	250	122	6	2.9%	-130.00 [-237.75, -22.25]
Zelai He combM109,2015	50	22	6	350	172	6	2.7%	-300.00 [-438.75, -161.25]
**Subtotal (95% CI)**			**154**			**146**	**40.5%**	**-328.64 [-416.05, -241.23]**
Heterogeneity: Tau² = 24183.99; Chi² = 66.59, df = 22 (P < 0.00001); I² = 67%								
Test for overall effect: Z = 7.37 (P < 0.00001)								
**2.2.2 Non Targeted DDS + Drug Vs Free Drug**								
Cao, 2016	480	238	4	500	248	4	1.5%	-20.00 [-356.85, 316.85]
Guo*,2019	400	197	6	700	347	6	1.6%	-300.00 [-619.28, 19.28]
He comb,2015	200	97	6	350	172	6	2.6%	-150.00 [-308.00, 8.00]
He combM109,2015	100	47	6	300	147	6	2.8%	-200.00 [-323.49, -76.51]
Iyer, 2020	343	168.5	6	1,372	683	6	0.8%	-1029.00 [-1591.89, -466.11]
Jin, 2016	150	70	10	1,350	670	10	1.2%	-1200.00 [-1617.52, -782.48]
Jin, 2020	250	121	8	820	406	8	1.7%	-570.00 [-863.57, -276.43]
Jung, 2012	190	92.5	5	250	122.5	5	2.8%	-60.00 [-194.55, 74.55]
Jung*,2012	200	97.5	5	220	107.5	5	2.8%	-20.00 [-147.21, 107.21]
Ling, 2015	640	316	8	690	341	8	1.6%	-50.00 [-372.16, 272.16]
Long,2014	400	198	4	500	273	4	1.5%	-100.00 [-430.49, 230.49]
Nguyen,2001	550	267	16	1,200	596	8	1.1%	-650.00 [-1083.23, -216.77]
Shi, 2023	350	170	10	500	245	10	2.4%	-150.00 [-334.82, 34.82]
Shuhang Wang, 2021	250	122	6	575	281.5	12	2.4%	-325.00 [-511.81, -138.19]
Song, 2014	150	72	6	200	97	6	3.0%	-50.00 [-146.66, 46.66]
Tang, 2010	80	37	6	750	372.5	5	1.6%	-670.00 [-997.84, -342.16]
Wang, 2018	800	396	8	950	471	8	1.1%	-150.00 [-576.41, 276.41]
Wang,2021	300	147	6	600	297	6	1.9%	-300.00 [-565.16, -34.84]
Wang,2025	580	287.5	5	550	272.5	5	1.5%	30.00 [-317.21, 377.21]
Wu, 2020	500	247	6	1,100	547	6	1.0%	-600.00 [-1080.24, -119.76]
Xionga, 2016	130	62	6	200	97	6	3.0%	-70.00 [-162.11, 22.11]
Yang, 2012	1,200	598.5	3	2,000	998.5	5	0.2%	-800.00 [-1906.64, 306.64]
Yang, 2023	350	172.5	5	500	247.5	5	1.9%	-150.00 [-414.43, 114.43]
Yuan, 2015	300	147.5	5	550	272.5	5	1.9%	-250.00 [-521.60, 21.60]
Yuan, 2016	252.6	121.3	10	270	130	10	2.9%	-17.40 [-127.60, 92.80]
Yu-Yang Bi, 2024	250	122.5	5	2,000	997.5	5	0.4%	-1750.00 [-2630.90, -869.10]
Zelai He comb,2015	150	72	6	250	122	6	2.9%	-100.00 [-213.35, 13.35]
Zelai He combM109,2015	120	57	6	350	172	6	2.7%	-230.00 [-374.99, -85.01]
Zhang,2018	200	97	6	700	347	6	1.8%	-500.00 [-788.30, -211.70]
Zhou,2013	150	72.5	5	300	147.5	5	2.7%	-150.00 [-294.06, -5.94]
Zhou, 2020	800	395	10	1,400	695	10	0.9%	-600.00 [-1095.47, -104.53]
Zhou*,2020	600	295	10	1,000	495	10	1.4%	-400.00 [-757.15, -42.85]
**Subtotal (95% CI)**			**214**			**213**	**59.5%**	**-244.91 [-317.72, -172.10]**
Heterogeneity: Tau² = 25008.02; Chi² = 113.26, df = 31 (P < 0.00001); I² = 73%								
Test for overall effect: Z = 6.59 (P < 0.00001)								
**Total (95% CI)**			**368**			**359**	**100.0%**	**-280.11 [-336.76, -223.46]**
Heterogeneity: Tau² = 25643.13; Chi² = 191.22, df = 54 (P < 0.00001); I² = 72%								
Test for overall effect: Z = 9.69 (P < 0.00001)								
Test for subgroup differences: Chi² = 2.08, df = 1 (P = 0.15), I² = 51.9%								

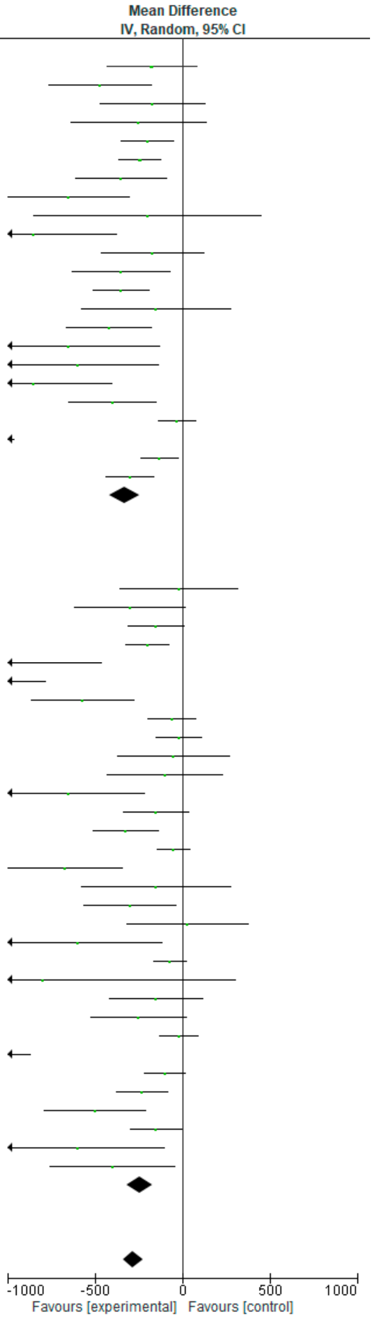

Figure S3. Efficacy of chemotherapeutics delivered via targeted and non-targeted drug delivery systems compared to free chemotherapeutics on tumor growth inhibition

Study or Subgroup	Targeted DDS+ Drug Mean	SD	Total	Non Targeted DDS + Drug Mean	SD	Total	Weight	Mean Difference IV, Random, 95% CI
Cao, 2016	250	121	8	480	238	4	2.8%	-230.00 [-477.85, 17.85]
Guo, 2019	230	112	6	300	147	6	5.8%	-70.00 [-217.87, 77.87]
He comb,2015	150	72	6	200	97	6	8.8%	-50.00 [-146.66, 46.66]
He combM109,2015	58	32	6	100	47	6	12.6%	-42.00 [-87.50, 3.50]
Li,2011	950	472.5	5	1,150	572.5	5	0.5%	-200.00 [-850.64, 450.64]
Li,2020	300	147	6	500	247	6	3.1%	-200.00 [-429.99, 29.99]
Ling, 2015	520	256	8	640	316	8	2.3%	-120.00 [-401.81, 161.81]
Long,2014	150	73	4	400	198	4	3.7%	-250.00 [-456.80, -43.20]
Shi, 2023	150	70	10	350	170	10	7.7%	-200.00 [-313.95, -86.05]
Wang, 2018	300	146	8	800	396	8	2.1%	-500.00 [-792.47, -207.53]
Wang,2025	130	62.5	5	580	287.5	5	2.6%	-450.00 [-707.89, -192.11]
Wu, 2020	250	122	6	500	247	6	3.4%	-250.00 [-470.43, -29.57]
Yuan, 2015	150	72.5	5	300	147.5	5	6.0%	-150.00 [-294.06, -5.94]
Yuan, 2016	238.5	114.25	10	252.6	121.3	10	8.3%	-14.10 [-117.38, 89.18]
Yu-Yang Bi, 2024	107	51	5	250	122.5	5	7.5%	-143.00 [-259.31, -26.69]
Zelai He comb,2015	120	57	6	150	72	6	10.5%	-30.00 [-103.48, 43.48]
Zelai He combM109,2015	50	22	6	120	57	6	12.4%	-70.00 [-118.89, -21.11]
**Total (95% CI)**			**110**			**106**	**100.0%**	**-118.13 [-164.28, -71.98]**

Heterogeneity: Tau² = 3866.62; Chi² = 36.23, df = 16 (P = 0.003); I² = 56%
Test for overall effect: Z = 5.02 (P < 0.00001)

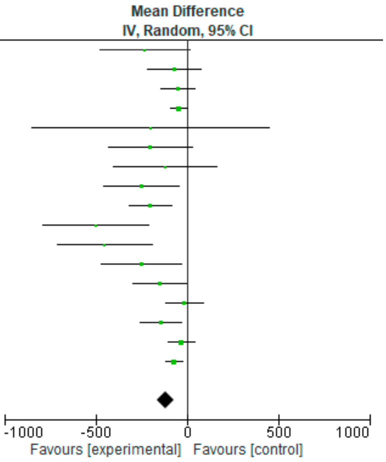

Figure S4. Efficacy of chemotherapeutics delivered via targeted versus non-targeted drug delivery systems on tumor growth inhibition

Study or Subgroup	DDS + Drug Mean	SD	Total	Free Drug Mean	SD	Total	Weight	Mean Difference IV, Random, 95% CI
**2.4.1 Cisplatin**								
Cao, 2016	326.7	157.35	12	500	248	4	3.0%	-173.30 [-432.13, 85.53]
He, 2015	450	22	6	620	307	6	3.1%	-170.00 [-416.28, 76.28]
He M109,2015	250	122	6	600	257	6	3.2%	-350.00 [-577.63, -122.37]
Iyer, 2020	343	168.5	6	1,372	683	6	1.7%	-1029.00 [-1591.89, -466.11]
Shuhang Wang, 2021	250	122	6	575	281	12	3.4%	-325.00 [-511.56, -138.44]
Wang,2025	130	62.5	5	550	272.5	5	3.1%	-420.00 [-665.05, -174.95]
Wang*,2018	700	346	8	1,350	671	8	1.8%	-650.00 [-1173.15, -126.85]
Wu, 2020	850	422	6	1,100	547	6	1.7%	-250.00 [-802.80, 302.80]
Xionga, 2016	130	62	6	200	97	6	3.7%	-70.00 [-162.11, 22.11]
Yang, 2023	350	172.5	5	500	247.5	5	3.0%	-150.00 [-414.43, 114.43]
Yu-Yang Bi, 2024	107	51	5	2,000	397.5	5	2.6%	-1893.00 [-2244.27, -1541.73]
Zelai He, 2015	300	147	6	550	272	6	3.1%	-250.00 [-497.39, -2.61]
Zelai He M109, 2015	250	122	6	600	297	6	3.0%	-350.00 [-606.91, -93.09]
Zhou, 2020	300	145	10	1,400	695	10	2.2%	-1100.00 [-1540.03, -659.97]
**Subtotal (95% CI)**			**93**			**91**	**38.6%**	**-481.20 [-691.12, -271.28]**
Heterogeneity: Tau² = 132857.48; Chi² = 127.40, df = 13 (P < 0.00001); I² = 90%								
Test for overall effect: Z = 4.49 (P < 0.00001)								
**2.4.2 Paclitaxel**								
Guo, 2019	265	126.5	12	600	297	6	3.1%	-335.00 [-583.19, -86.81]
Guo*,2019	400	197	6	700	347	6	2.7%	-300.00 [-619.28, 19.28]
He*,2015	550	272	6	800	397	6	2.4%	-250.00 [-635.07, 135.07]
He M109*,2015	200	97	6	850	422	6	2.6%	-650.00 [-996.47, -303.53]
Jung*,2012	200	97.5	5	220	107.5	5	3.6%	-20.00 [-147.21, 107.21]
Li,2011	950	472.5	5	1,150	572.5	5	1.4%	-200.00 [-850.64, 450.64]
Nguyen,2001	550	267	16	1,200	596	8	2.2%	-650.00 [-1083.23, -216.77]
Shi, 2023	150	70	10	500	245	10	3.5%	-350.00 [-507.93, -192.07]
Song*,2014	300	147	6	400	197	6	3.3%	-100.00 [-296.68, 96.68]
Wang**,2018	600	296	8	1,200	596	8	2.1%	-600.00 [-1061.13, -138.87]
Yang, 2012	1,200	598.5	3	2,000	998.5	3	0.5%	-800.00 [-2117.32, 517.32]
Yuan, 2015	225	107.5	10	550	272.5	5	3.1%	-325.00 [-572.97, -77.03]
Yuan, 2016	248.25	114.125	20	270	130	10	3.7%	-21.75 [-116.59, 73.09]
Zelai He*,2015	400	197	6	750	372	6	2.6%	-350.00 [-686.82, -13.18]
Zelai He M109*,2015	300	147	6	900	447	6	2.5%	-600.00 [-976.51, -223.49]
Zhang,2018	375	181.5	12	700	347	6	2.8%	-325.00 [-621.03, -28.97]
Zhou,2013	150	72.5	5	300	147.5	5	3.5%	-150.00 [-294.06, -5.94]
**Subtotal (95% CI)**			**142**			**107**	**45.7%**	**-281.05 [-385.11, -176.99]**
Heterogeneity: Tau² = 25194.72; Chi² = 47.75, df = 16 (P < 0.0001); I² = 66%								
Test for overall effect: Z = 5.29 (P < 0.00001)								
**2.4.3 Docetaxel**								
Jin, 2016	150	70	10	1,350	670	10	2.3%	-1200.00 [-1617.52, -782.48]
Jung, 2012	190	92.5	5	250	122.5	5	3.6%	-60.00 [-194.55, 74.55]
Li,2020	275	131.5	12	1,150	572	6	2.1%	-875.00 [-1338.69, -411.31]
Ling, 2015	520	256	8	690	341	8	2.8%	-170.00 [-465.47, 125.47]
Wang,2021	300	147	6	600	297	6	3.0%	-300.00 [-565.16, -34.84]
Wu*,2020	550	272	6	1,100	547	6	2.0%	-550.00 [-1038.81, -61.19]
**Subtotal (95% CI)**			**47**			**41**	**15.7%**	**-488.12 [-817.96, -158.27]**
Heterogeneity: Tau² = 137629.42; Chi² = 36.34, df = 5 (P < 0.00001); I² = 86%								
Test for overall effect: Z = 2.90 (P = 0.004)								
**Total (95% CI)**			**282**			**239**	**100.0%**	**-390.39 [-489.25, -291.53]**
Heterogeneity: Tau² = 66630.68; Chi² = 217.31, df = 36 (P < 0.00001); I² = 83%								
Test for overall effect: Z = 7.74 (P < 0.00001)								
Test for subgroup differences: Chi² = 3.72, df = 2 (P = 0.16), I² = 46.3%								

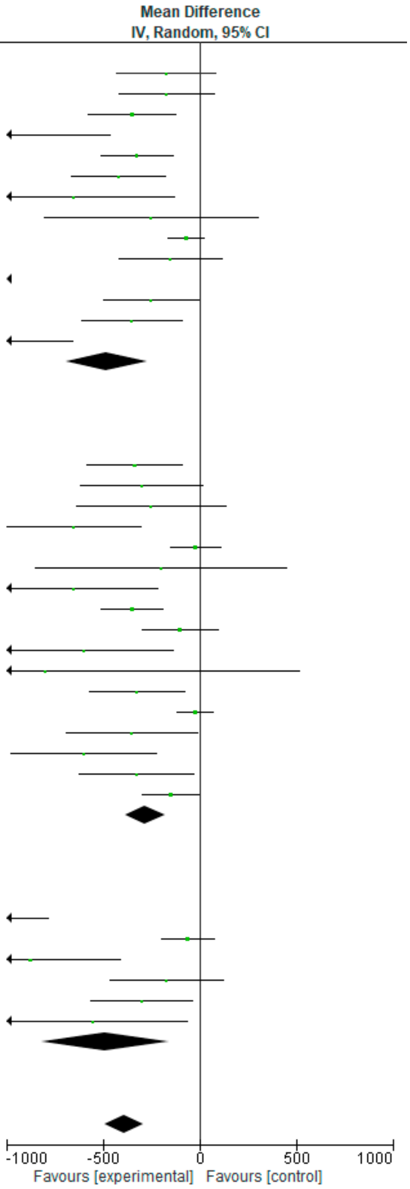

Figure S5. Efficacy of chemotherapeutics delivered via drug delivery systems compared to free chemotherapeutics on tumor growth inhibition: Subgroup analysis of Cisplatin, Paclitaxel, and Docetaxel.

Study or Subgroup	Two Cytostatic Drugs Mean	SD	Total	Two Free Drugs Mean	SD	Total	Weight	Mean Difference IV, Random, 95% CI
**2.5.1 CIsplatin +Paclitaxel**								
He comb,2015	175	81.5	12	350	172	6	15.4%	-175.00 [-320.15, -29.85]
He combM109,2015	79	33.5	12	300	147	6	16.9%	-221.00 [-340.14, -101.86]
Song, 2014	150	72	6	200	97	6	18.2%	-50.00 [-146.66, 46.66]
Wang, 2018	300	146	8	950	471	8	7.0%	-650.00 [-991.70, -308.30]
Zelai He comb,2015	135	61.5	12	250	122	6	17.8%	-115.00 [-218.63, -11.37]
Zelai He combM109,2015	85	36.5	12	350	172	6	15.8%	-265.00 [-404.17, -125.83]
**Subtotal (95% CI)**			**62**			**38**	**91.1%**	**-191.93 [-292.53, -91.33]**
Heterogeneity: Tau² = 10296.10; Chi² = 16.82, df = 5 (P = 0.005); I² = 70%								
Test for overall effect: Z = 3.74 (P = 0.0002)								
**2.5.2 Cisplatin + Docetaxel**								
Wu, 2020	500	247	6	1,100	547	6	4.2%	-600.00 [-1080.24, -119.76]
Wu*,2020	433	207.5	18	1,100	547	6	4.7%	-667.00 [-1115.06, -218.94]
**Subtotal (95% CI)**			**24**			**12**	**8.9%**	**-635.82 [-963.43, -308.21]**
Heterogeneity: Tau² = 0.00; Chi² = 0.04, df = 1 (P = 0.84); I² = 0%								
Test for overall effect: Z = 3.80 (P = 0.0001)								
**Total (95% CI)**			**86**			**50**	**100.0%**	**-237.88 [-348.20, -127.56]**
Heterogeneity: Tau² = 15023.36; Chi² = 24.88, df = 7 (P = 0.0008); I² = 72%								
Test for overall effect: Z = 4.23 (P < 0.0001)								
Test for subgroup differences: Chi² = 6.44, df = 1 (P = 0.01), I² = 84.5%								

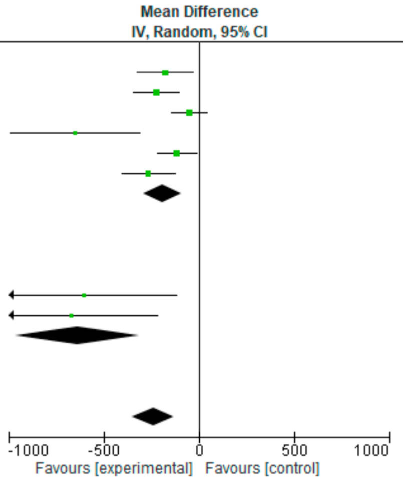

Figure S6. Efficacy of combination of two chemotherapeutics delivered via drug delivery systems compared to free chemotherapeutics on tumor growth inhibition: Subgroup analysis of combination of two cytostatic drugs Cisplatin+ Paclitaxel, and Cisplatin + Docetaxel

Study or Subgroup	DDS+ Drug Mean	DDS+ Drug SD	DDS+ Drug Total	Free Drug Mean	Free Drug SD	Free Drug Total	Weight	Mean Difference IV, Random, 95% CI
**2.6.1 Intraperitoneal**								
Ling, 2015	580	282	16	690	341	8	2.5%	-110.00 [-383.73, 163.73]
Nguyen,2001	550	267	16	1,200	596	8	1.5%	-650.00 [-1083.23, -216.77]
**Subtotal (95% CI)**			**32**			**16**	**4.0%**	**-352.82 [-879.32, 173.68]**
Heterogeneity: Tau² = 111618.53; Chi² = 4.27, df = 1 (P = 0.04); I² = 77%								
Test for overall effect: Z = 1.31 (P = 0.19)								
**2.6.2 Intravenous**								
Cao, 2016	326.7	157.35	12	500	248	4	2.6%	-173.30 [-432.13, 85.53]
Guo, 2019	265	126.5	12	600	297	6	2.7%	-335.00 [-583.19, -86.81]
Guo*,2019	400	197	6	700	347	6	2.2%	-300.00 [-619.28, 19.28]
He, 2015	450	222	6	620	307	6	2.3%	-170.00 [-473.14, 133.14]
He*,2015	550	272	6	800	397	6	1.7%	-250.00 [-635.07, 135.07]
He comb,2015	150	72	6	350	172	6	3.7%	-200.00 [-349.20, -50.80]
He combM109,2015	58	32	6	300	147	6	3.9%	-242.00 [-362.38, -121.62]
He M109,2015	250	122	6	600	297	6	2.6%	-350.00 [-606.91, -93.09]
He M109*,2015	200	97	6	850	422	6	2.0%	-650.00 [-996.47, -303.53]
Iyer, 2020	343	168.5	6	1,375	683	6	1.0%	-1032.00 [-1594.89, -469.11]
Jin, 2020	425	196.5	32	820	406	8	2.4%	-395.00 [-684.46, -105.54]
Jung, 2012	190	92.5	5	250	122.5	5	3.8%	-60.00 [-194.55, 74.55]
Jung*,2012	200	97.5	5	220	107.5	5	3.9%	-20.00 [-147.21, 107.21]
Li,2011	950	472.5	5	1,150	572.5	5	0.8%	-200.00 [-850.64, 450.64]
Li,2020	275	131.5	12	1,150	572	6	1.4%	-875.00 [-1338.69, -411.31]
Shi, 2023	150	70	10	500	245	10	3.6%	-350.00 [-507.93, -192.07]
Shuhang Wang, 2021	250	122	6	575	281.5	12	3.3%	-325.00 [-511.81, -138.19]
Wang, 2018	300	146	8	950	471	8	2.0%	-650.00 [-991.70, -308.30]
Wang,2021	300	147	6	600	297	6	2.6%	-300.00 [-565.16, -34.84]
Wang*,2018	700	346	8	1,350	671	8	1.1%	-650.00 [-1173.15, -126.85]
Wang**,2018	600	296	8	1,200	596	8	1.4%	-600.00 [-1061.13, -138.87]
Wu, 2020	375	181.5	12	1,100	547	6	1.4%	-725.00 [-1174.57, -275.43]
Xionga, 2016	130	62	6	200	97	6	4.2%	-70.00 [-162.11, 22.11]
Yang, 2012	1,200	598.5	3	2,000	998.5	3	0.2%	-800.00 [-2117.32, 517.32]
Yang, 2023	350	172.5	5	500	247.5	5	2.6%	-150.00 [-414.43, 114.43]
Yuan, 2015	150	72.5	5	300	147.5	5	3.7%	-150.00 [-294.06, -5.94]
Yuan, 2016	248.25	114.125	20	270	130	10	4.2%	-21.75 [-116.59, 73.09]
Yu-Yang Bi, 2024	107	51	5	1,900	947.5	5	0.5%	-1793.00 [-2624.71, -961.29]
Zelai He, 2015	300	147	6	550	272	6	2.7%	-250.00 [-497.39, -2.61]
Zelai He*,2015	400	197	6	750	372	6	2.0%	-350.00 [-686.82, -13.18]
Zelai He comb,2015	135	61.5	12	250	122	6	4.1%	-115.00 [-218.63, -11.37]
Zelai He combM109,2015	85	36.5	12	350	172	6	3.8%	-265.00 [-404.17, -125.83]
Zelai He M109, 2015	250	122	6	600	297	6	2.6%	-350.00 [-606.91, -93.09]
Zelai He M109*,2015	300	147	6	900	447	6	1.8%	-600.00 [-976.51, -223.49]
Zhang,2018	375	181.5	12	700	347	6	2.3%	-325.00 [-621.03, -28.97]
Zhou, 2020	800	395	10	1,400	695	10	1.2%	-600.00 [-1095.47, -104.53]
Zhou*,2020	600	295	10	1,000	495	10	1.9%	-400.00 [-757.15, -42.85]
**Subtotal (95% CI)**			**313**			**241**	**90.2%**	**-297.30 [-364.50, -230.10]**
Heterogeneity: Tau² = 22983.28; Chi² = 114.33, df = 36 (P < 0.00001); I² = 69%								
Test for overall effect: Z = 8.67 (P < 0.00001)								
**2.6.3 Intratumoral**								
Tang, 2010	80	37	6	750	372.5	5	2.1%	-670.00 [-997.84, -342.16]
Zhou,2013	150	72.5	5	300	147.5	5	3.7%	-150.00 [-294.06, -5.94]
**Subtotal (95% CI)**			**11**			**10**	**5.8%**	**-388.29 [-896.10, 119.52]**
Heterogeneity: Tau² = 118509.04; Chi² = 8.10, df = 1 (P = 0.004); I² = 88%								
Test for overall effect: Z = 1.50 (P = 0.13)								
**Total (95% CI)**			**356**			**267**	**100.0%**	**-301.03 [-365.35, -236.70]**
Heterogeneity: Tau² = 23560.21; Chi² = 127.38, df = 40 (P < 0.00001); I² = 69%								
Test for overall effect: Z = 9.17 (P < 0.00001)								
Test for subgroup differences: Chi² = 0.16, df = 2 (P = 0.92), I² = 0%								

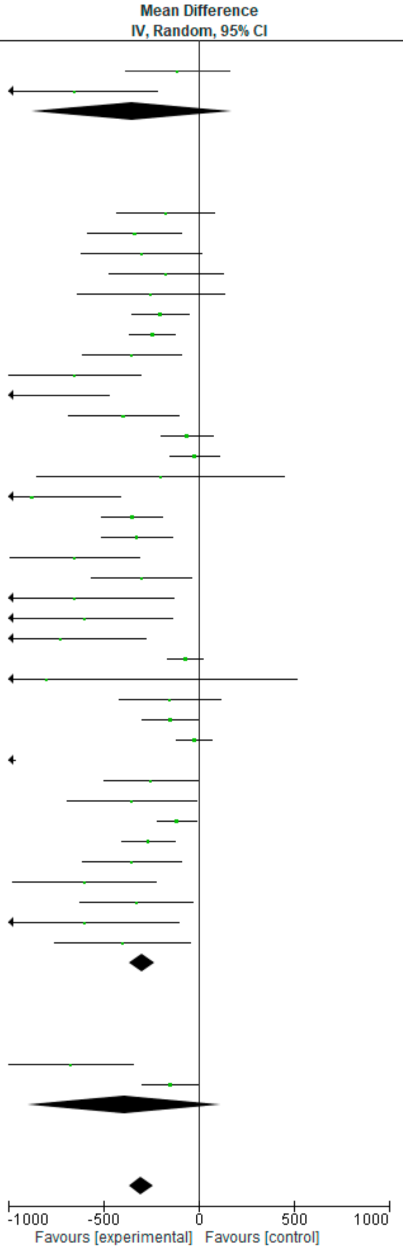

Figure S7. Efficacy of chemotherapeutics in DDS compared to free chemotherapeutics on tumor growth inhibition in subgroup analysis: Intravenous, Intraperitoneal, and intratumoral.

Study or Subgroup	DDS+ Drug Mean	DDS+ Drug SD	DDS+ Drug Total	Free Drug Mean	Free Drug SD	Free Drug Total	Weight	Mean Difference IV, Random, 95% CI
**2.7.1 Nanoparticles**								
He, 2015	450	222	6	620	307	6	2.1%	-170.00 [-473.14, 133.14]
He*,2015	550	272	6	800	397	6	1.7%	-250.00 [-635.07, 135.07]
He comb,2015	175	81.5	12	350	172	6	2.9%	-175.00 [-320.15, -29.85]
He combM109,2015	79	33.5	12	300	147	6	3.0%	-221.00 [-340.14, -101.86]
He M109,2015	250	122	6	600	297	6	2.3%	-350.00 [-606.91, -93.09]
He M109*,2015	200	97	6	850	422	6	1.9%	-650.00 [-996.47, -303.53]
Iyer, 2020	343	168.5	6	1,372	683	6	1.1%	-1029.00 [-1591.89, -466.11]
Jin, 2016	150	70	10	1,350	670	10	1.6%	-1200.00 [-1617.52, -782.48]
Jin, 2020	425	196.5	32	820	406	8	2.2%	-395.00 [-684.46, -105.54]
Jung, 2012	190	92.5	5	250	122.5	5	2.9%	-60.00 [-194.55, 74.55]
Jung*,2012	200	97.5	5	220	107.5	5	3.0%	-20.00 [-147.21, 107.21]
Li,2011	950	472.5	5	1,150	572.5	5	0.9%	-200.00 [-850.64, 450.64]
Li,2020	275	131.5	12	1,150	572	6	1.4%	-875.00 [-1338.69, -411.31]
Ling, 2015	580	282	16	690	341	8	2.2%	-110.00 [-383.73, 163.73]
Long,2014	275	133.5	8	500	248	4	2.3%	-225.00 [-485.05, 35.05]
Shi, 2023	250	115	20	500	245	10	2.8%	-250.00 [-410.00, -90.00]
Tang, 2010	80	37	6	750	372.5	5	2.0%	-670.00 [-997.84, -342.16]
Wu, 2020	375	181.5	12	1,100	547	6	1.5%	-725.00 [-1174.57, -275.43]
Yang, 2012	1,200	598.5	3	2,000	998.5	3	0.3%	-800.00 [-2117.32, 517.32]
Yang, 2023	200	95	10	500	247.5	5	2.5%	-300.00 [-524.79, -75.21]
Yuan, 2016	248.25	114.125	20	270	130	10	3.1%	-21.75 [-116.59, 73.09]
Yu-Yang Bi, 2024	178.5	84.25	10	2,000	397.5	5	1.9%	-1821.50 [-2173.81, -1469.19]
Zelai He, 2015	300	147	6	550	272	6	2.4%	-250.00 [-497.39, -2.61]
Zelai He*,2015	400	197	6	750	372	6	1.9%	-350.00 [-686.82, -13.18]
Zelai He comb,2015	135	61.5	12	250	122	6	3.1%	-115.00 [-218.63, -11.37]
Zelai He combM109,2015	85	36.5	12	350	172	6	2.9%	-265.00 [-404.17, -125.83]
Zelai He M109, 2015	250	122	6	600	297	6	2.3%	-350.00 [-606.91, -93.09]
Zelai He M109*,2015	300	147	6	900	447	6	1.8%	-600.00 [-976.51, -223.49]
Zhou,2013	150	72.5	5	300	147.5	5	2.9%	-150.00 [-294.06, -5.94]
Zhou, 2020	800	395	10	1,400	695	10	1.3%	-600.00 [-1095.47, -104.53]
Zhou*,2020	600	695	10	1,400	695	10	1.0%	-800.00 [-1409.18, -190.82]
**Subtotal (95% CI)**			**301**			**198**	**65.2%**	**-382.71 [-483.70, -281.73]**
Heterogeneity: Tau² = 57155.11; Chi² = 187.01, df = 30 (P < 0.00001); I² = 84%								
Test for overall effect: Z = 7.43 (P < 0.00001)								
**2.7.2 Liposomes**								
Cao, 2016	326.7	157.35	12	500	248	4	2.3%	-173.30 [-432.13, 85.53]
Guo, 2019	346	161	24	700	347	6	2.2%	-354.00 [-639.03, -68.97]
Guo*,2019	400	197	6	700	347	6	2.0%	-300.00 [-619.28, 19.28]
Nguyen,2001	550	267	16	1,200	596	8	1.5%	-650.00 [-1083.23, -216.77]
Wang, 2018	300	146	8	950	471	8	1.9%	-650.00 [-991.70, -308.30]
Wang,2025	410	197.5	15	550	272.5	5	2.3%	-140.00 [-398.92, 118.92]
Wang*,2018	700	346	8	1,350	671	8	1.2%	-650.00 [-1173.15, -126.85]
Wang**,2018	600	296	8	1,200	596	8	1.4%	-600.00 [-1061.13, -138.87]
Wu, 2020	375	181.5	12	1,100	547	6	1.5%	-725.00 [-1174.57, -275.43]
Xionga, 2016	130	62	6	200	97	6	3.1%	-70.00 [-162.11, 22.11]
**Subtotal (95% CI)**			**115**			**65**	**19.5%**	**-376.33 [-547.61, -205.04]**
Heterogeneity: Tau² = 47171.32; Chi² = 31.14, df = 9 (P = 0.0003); I² = 71%								
Test for overall effect: Z = 4.31 (P < 0.0001)								
**2.7.3 Micelles**								
Shuhang Wang, 2021	250	122	6	575	281	12	2.7%	-325.00 [-511.56, -138.44]
Song, 2014	150	72	6	200	97	6	3.1%	-50.00 [-146.66, 46.66]
Song*,2014	300	147	6	400	197	6	2.6%	-100.00 [-296.68, 96.68]
Wang,2021	300	147	6	600	297	6	2.3%	-300.00 [-565.16, -34.84]
Yuan, 2015	225	107.5	10	550	272.5	5	2.4%	-325.00 [-572.97, -77.03]
Zhang,2018	375	181.5	12	700	347	6	2.1%	-325.00 [-621.03, -28.97]
**Subtotal (95% CI)**			**46**			**41**	**15.2%**	**-211.45 [-337.99, -84.92]**
Heterogeneity: Tau² = 13733.43; Chi² = 12.22, df = 5 (P = 0.03); I² = 59%								
Test for overall effect: Z = 3.28 (P = 0.001)								
**Total (95% CI)**			**462**			**304**	**100.0%**	**-349.71 [-423.59, -275.83]**
Heterogeneity: Tau² = 43744.76; Chi² = 232.69, df = 46 (P < 0.00001); I² = 80%								
Test for overall effect: Z = 9.28 (P < 0.00001)								
Test for subgroup differences: Chi² = 4.69, df = 2 (P = 0.10), I² = 57.4%								

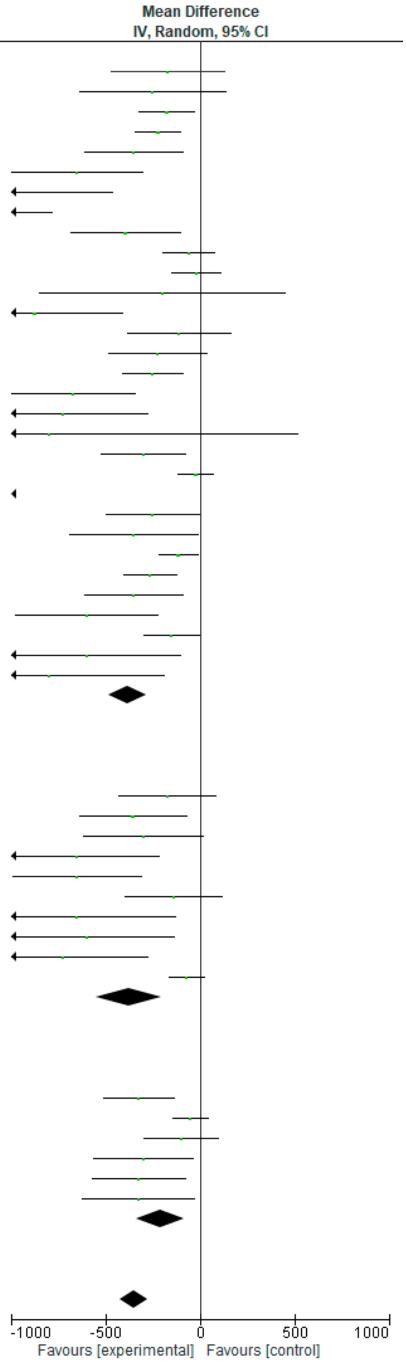

Figure S8. Efficacy of chemotherapeutics in various DDS types compared to free chemotherapeutics on tumor growth inhibition in subgroup analysis: Nanoparticles, Liposomes, and Micelles

Table S4. Assessment of risk of bias in the included studies using SYRCLE's RoB tool for animal studies.

Nr.	References	1. Is it mentioned that the experiment was randomized?	2. Is it mentioned that the experiment was blinded (level unknown)?	3. Is a power/sample size calculation shown?	4. Was the allocation sequence adequately generated and applied?	5. Was the allocation adequately concealed?	6. Were the caregivers/ and or investigators during the course of the experiment blinded from knowledge of which intervention each animal received?	7. Was the outcome assessor blinded?	8. Are the animals randomly housed during the experiment?	9. Were animals selected at random for the outcome assessment?	10. Were the groups similar at baseline or was adjusted for confounders in the analysis?	11. Were incomplete outcome data adequately addressed?	12. Was the study apparently free of other problems that could pose a high risk of bias?
1	Cao 2015 (28)	Y	N	N	N	U	U	U	U	U	Y	Y	Y
2	Guo 2019 (29)	Y	N	N	N	U	U	U	U	U	Y	Y	Y
3	He 2015 (30)	U	N	U	U	U	U	U	U	U	Y	Y	Y
4	Iyer 2020 (31)	Y	N	N	N	U	U	U	U	U	Y	Y	Y
5	Jin 2016 (32)	U	N	Y	N	U	U	U	U	U	Y	Y	Y
6	Jin 2020 (33)	Y	N	N	N	U	U	U	U	U	Y	Y	Y
7	Jung 2012 (34)	Y	N	N	N	U	U	U	U	U	Y	Y	Y
8	Li 2011 (35)	N	N	N	U	U	U	U	U	U	Y	Y	Y
9	Li 2020 (36)	Y	N	N	N	U	U	U	U	U	Y	Y	Y
10	Ling, 2015 (37)	Y	N	N	U	U	U	U	U	U	Y	Y	Y
11	Long 2014 (38)	N	N	N	U	U	U	U	U	U	Y	Y	Y
12	Nguyen, 2001(39)	N	N	N	U	U	U	U	U	U	Y	Y	Y
13	Shi 2023 (40)	Y	U	N	N	U	U	U	U	U	Y	Y	Y
14	Shuhang Wang 2021 (41)	Y	N	N	U	U	U	U	U	U	Y	Y	Y
15	Song 2014 (42)	Y	N	N	U	U	U	U	U	U	Y	Y	Y
16	Tang 2010 (43)	N	N	N	U	U	U	U	U	U	Y	Y	Y
17	Wang 2018 (44)	Y	N	N	U	U	U	U	U	U	Y	Y	Y
18	Wang 2021 (45)	Y	N	N	U	U	U	U	U	U	Y	Y	Y
19	Wang 2025 (46)	Y	N	N	U	U	U	U	U	U	Y	Y	Y
20	Wu 2020 (47)	Y	N	N	U	U	U	U	U	U	Y	Y	Y
21	Xiong 2016 (48)	Y	N	N	U	U	U	U	U	U	Y	Y	Y
22	Yang 2012 (49)	Y	N	N	U	U	U	U	U	U	Y	Y	Y
23	Yang 2023 (50)	Y	N	N	U	U	U	U	U	U	Y	Y	Y
24	Yuan 2015 (51)	N	N	N	U	U	U	U	U	U	Y	Y	Y
25	Yuan 2016 (52)	Y	N	N	U	U	U	U	U	U	Y	Y	Y

26	Yu Yang Bi 2024 (53)	Y	N	N	U	U	U	U	U	U	Y	Y	Y
27	Zelai He 2015 (54)	Y	N	N	U	U	U	U	U	U	Y	Y	Y
28	Zhang 2018 (55)	Y	N	N	U	U	U	U	U	U	Y	Y	Y
29	Zhou 2013 (56)	Y	N	N	U	U	U	U	U	U	Y	Y	Y
30	Zhou 2020 (57)	Y	N	N	U	U	U	U	U	U	Y	Y	Y
**Yes (Low)**		76.7%	0%	3.3%	0%	0%	0%	0%	0%	0%	100%	100%	100%
**Unclear**		6.7%	3.3%	3.3%	73.3%	100%	100%	100%	100%	100%	0%	0%	0%
**No (High)**		16.7%	96.7%	93.3%	26.7%	0%	0%	0%	0%	0%	0%	0%	0%

Yes (L) Low risk of bias; No (H) High risk of bias; Unclear (U) If insufficient details have been reported to assess the risk of bias properly.
